# Functional and genomic characterisation of a xenograft model system for the study of metastasis in triple-negative breast cancer

**DOI:** 10.1242/dmm.032250

**Published:** 2018-05-29

**Authors:** Cameron N. Johnstone, Andrew D. Pattison, Kylie L. Gorringe, Paul F. Harrison, David R. Powell, Peter Lock, David Baloyan, Matthias Ernst, Alastair G. Stewart, Traude H. Beilharz, Robin L. Anderson

**Affiliations:** 1 Cancer Research Division, Peter MacCallum Cancer Centre, Victorian Comprehensive Cancer Centre, Parkville, Victoria 3050, Australia; 2 Sir Peter MacCallum Department of Oncology, University of Melbourne, Parkville, Victoria 3050, Australia; 3 Department of Pathology, University of Melbourne, Parkville, Victoria 3010, Australia; 4 Olivia Newton-John Cancer Research Institute, Heidelberg, Victoria 3084, Australia; 5 School of Cancer Medicine, La Trobe University, Bundoora, Victoria 3086, Australia; 6 Department of Biochemistry and Molecular Biology, Monash University, Clayton, Victoria 3800, Australia; 7 Department of Pharmacology & Therapeutics, University of Melbourne, Parkville, Victoria 3010, Australia; 8 Biomedicine Discovery Institute, Monash University, Clayton, Victoria 3800, Australia; 9 Monash Bioinformatics Platform, Monash University, Clayton, Victoria 3800, Australia; 10 LIMS Bioimaging Facility, La Trobe Institute for Molecular Science, La Trobe University, Bundoora, Victoria 3086, Australia

**Keywords:** Triple-negative, Breast cancer, Metastasis, Mouse model, Xenograft

## Abstract

Triple-negative breast cancer (TNBC) represents 10-20% of all human ductal adenocarcinomas and has a poor prognosis relative to other subtypes. Hence, new molecular targets for therapeutic intervention are necessary. Analyses of panels of human or mouse cancer lines derived from the same individual that differ in their cellular phenotypes but not in genetic background have been instrumental in defining the molecular players that drive the various hallmarks of cancer. To determine the molecular regulators of metastasis in TNBC, we completed a rigorous *in vitro* and *in vivo* characterisation of four populations of the MDA-MB-231 human breast cancer line ranging in aggressiveness from non-metastatic to spontaneously metastatic to lung, liver, spleen and lymph node. Single nucleotide polymorphism (SNP) array analyses and genome-wide mRNA expression profiles of tumour cells isolated from orthotopic mammary xenografts were compared between the four lines to define both cell autonomous pathways and genes associated with metastatic proclivity. Gene set enrichment analysis (GSEA) demonstrated an unexpected association between both ribosome biogenesis and mRNA metabolism and metastatic capacity. Differentially expressed genes or families of related genes were allocated to one of four categories, associated with either metastatic initiation (e.g. *CTSC*, *ENG*, *BMP2*), metastatic virulence (e.g. *ADAMTS1*, *TIE1*), metastatic suppression (e.g. *CST1*, *CST2*, *CST4*, *CST6*, *SCNNA1*, *BMP4*) or metastatic avirulence (e.g. *CD74*). Collectively, this model system based on MDA-MB-231 cells should be useful for the assessment of gene function in the metastatic cascade and also for the testing of novel experimental therapeutics for the treatment of TNBC.

This article has an associated First Person interview with the first author of the paper.

## Abstract (precis)

**Summary:** Four MDA-MB-231 cell line variants were evaluated for spontaneous metastatic capacity. DNA SNP arrays and RNA-Seq analysis of xenografted tumour cells were conducted to identify genes associated with metastatic proclivity.

## INTRODUCTION

Genetic and genomic analyses have shown that mammary ductal adenocarcinoma, the major form of breast cancer in humans, clusters into several distinct subtypes that are associated with significant differences in patient mortality rates (Curtis et al., 2012; Perou et al., 2000; Sorlie et al., 2001). Unlike hormone-receptor-positive tumours that express the oestrogen receptor (ER) and progesterone receptor (PR), or HER-2-positive neoplasms that express the erb-b2 transmembrane receptor, triple-negative breast cancers (TNBCs) are negative for all three receptors (Nielsen et al., 2004; Prat et al., 2013). Since approved targeted therapies are lacking, the standard treatment modalities for TNBC are adjuvant radio- and chemotherapy following surgery. Despite aggressive intervention, TNBC displays the worst outcome of all breast cancer subtypes due to its propensity for early relapse and the development of resistance to chemotherapeutic drugs (Carey et al., 2007; Eckhardt et al., 2012; Liedtke et al., 2008). The discovery of new molecular targets for TNBC is also complicated by considerable inter- and intra-tumour heterogeneity. Indeed, genome-wide expression and DNA copy number analyses have described at least four distinct variants (Hennessy et al., 2009; Lehmann et al., 2011, 2016; Prat et al., 2010; Turner and Reis-Filho, 2013).

To discover the cellular alterations that lead to acquisition of intrinsic malignant cancer cell phenotypes, such as unlimited growth potential, metabolic rewiring, invasive capacity, metastatic ability and chemoresistance (Hanahan and Weinberg, 2011), analyses of panels of congenic breast cancer lines derived from the same individual but differing in these cellular phenotypes have been conducted (Aslakson and Miller, 1992; Chang et al., 2007; Eckhardt et al., 2005; Halldorsson et al., 2017; Johnstone et al., 2015; Minn et al., 2005b; Morris et al., 1993; Santner et al., 2001; Weaver et al., 1995). This has led not only to the elucidation of gene networks underlying these processes, but also to potential new molecular targets for therapy (Eckhardt et al., 2005; Johnstone et al., 2015; Minn et al., 2005a).

Here, we comprehensively characterised the orthotopic growth and metastatic ability of four different variants of the human TNBC cell line MDA-MB-231, originally isolated from the pleural effusion of a patient with metastatic breast cancer (Cailleau et al., 1974). Genome-wide expression profiling was completed by RNA-sequencing (RNA-Seq) on primary tumour cells isolated from non-metastatic MDA-MB-231_ATCC (231_ATCC) (Cailleau et al., 1974), moderately metastatic MDA-MB-231_LNA (231_LNA) and highly-metastatic MDA-MB-231_LM2 (231_LM2) (Minn et al., 2005a), and MDA-MB-231_HM.LNm5 (231_HM.LNm5) (Chang et al., 2007, 2008; Fietz et al., 2017) mammary xenografts. The DNA content and genomic DNA copy number alterations were also analysed in each of the four lines. Differentially expressed genes or gene families were allocated to one of four categories, associated with either metastatic initiation, metastatic virulence, metastatic suppression or metastatic avirulence (Nguyen et al., 2009).

## RESULTS

### Characterisation of spontaneous metastatic potential of MDA-MB-231-derived lines

To identify genes involved in progression and metastasis of human TNBC, we firstly comprehensively characterised four human breast cancer xenograft models for differences in spontaneous metastatic capacity. Each tumour line was derived from MDA-MB-231 human TNBC cells (Fig. S1), originally isolated from the pleural effusion of a patient with metastatic breast cancer (Cailleau et al., 1974). Each of the four lines was shown to match MDA-MB-231 by short tandem repeat (STR) profiling (see Materials and Methods). 231_ATCC is an early-passage line (passage number<20). The 231_I line is a population of MDA-MB-231 cells subcultured more than 50 times *in vitro* (Mongroo et al., 2004), and its daughter line, 231_LNA, was isolated from a lymph node metastasis that developed from a 231_I primary orthotopic tumour (Fig. S1, Table S1). 231_LM2 cells were isolated from an experimental lung metastasis that arose in mice inoculated with a reporter-gene-tagged version of the parental line, and have been extensively characterised elsewhere (Minn et al., 2005a). Lastly, 231_HM.LNm5 cells were isolated in our laboratory from a lymph node metastasis in a mouse bearing a MDA-MB-231HM primary mammary tumour (Fietz et al., 2017). The MDA-MB-231HM line was originally isolated from a spontaneous lung metastasis that arose following six rounds of *in vivo* passaging, whereby spontaneous secondary lesions forming in the lung were isolated and expanded *ex vivo* and subsequently re-implanted into the mammary gland (Chang et al., 2007, 2008). We and others have documented their aggressive metastatic phenotype in mice (Chang et al., 2015; Fietz et al., 2017; Jin et al., 2012; Le et al., 2016).

**Fig. 1.**
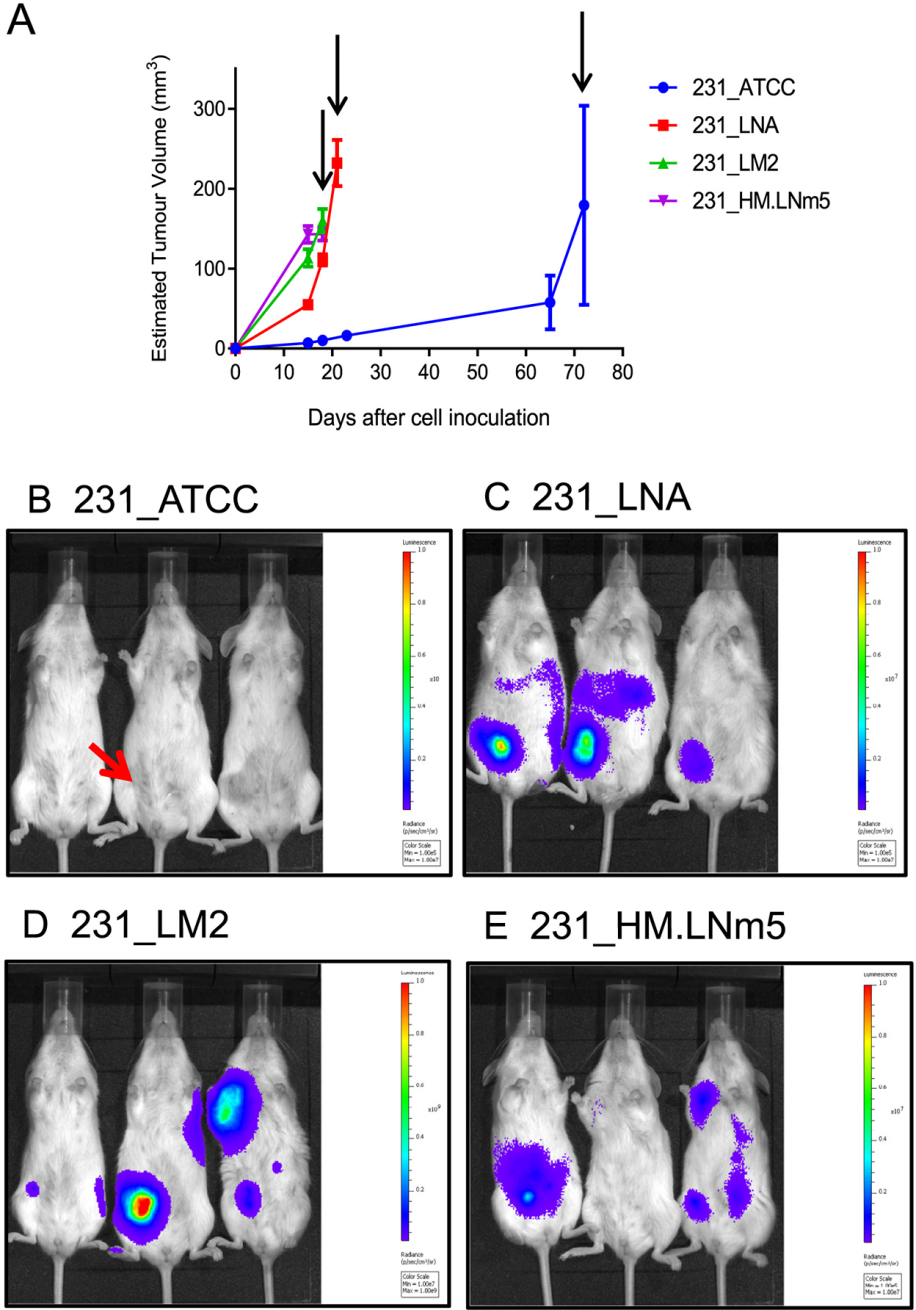
**Comparison of four MDA-MB-231-derived tumour variants *in vivo*.** (A) Primary-tumour growth rates were measured using electronic callipers. Mean tumour volume±s.e.m. is shown. 231_ATCC (*n*=3), 231_LNA (*n*=4), 231_LM2 (*n*=4), 231_HM.LNm5 (*n*=4). Differences in growth rates were determined using mixed-effects linear regression modelling (Johnstone et al., 2015): 231_ATCC vs 231_LNA (*P*=0.002), 231_ATCC vs 231_LM2 (*P*=0.002), 231_ATCC vs 231_HM (*P*=0.001), 231_LNA vs 231_LM2 (*P*=0.006), 231_LNA vs 231_HM (*P*=0.002), 231_LM2 vs 231_HM (*P*=0.168). 231_LM2 and 231_HM.LNm5 primary tumours were surgically resected at day 18, 231_LNA at day 21 and 231_ATCC at day 72 after inoculation (arrows). (B-E) *In vivo* bioluminescence imaging of breast cancer xenograft models. Luciferase images of live mice were captured 22 days following surgical resection of the primary mammary tumour for all models. Both local and distant tumour recurrence was present in each of the three metastatic models [(C) 231_LNA, (D) 231_LM2, (E) 231_HM.LNm5] but not in mice inoculated with 231_ATCC cells (B). Three mice are shown per model [*n*=4 for each model except for 231_ATCC (*n*=3)]. 231_LM2 cells express Firefly luciferase-2 (luc2), radiance scale 1×10^7^ (min) to 1×10^9^ (max). The other three models express the dimmer Firefly luciferase-1 (luc1), radiance scales 1×10^5^ (min) to 1×10^7^ (max). The site of original primary tumour formation in the right-side inguinal mammary gland is indicated with a red arrow in one mouse as an example (B).

**Fig. 2.**
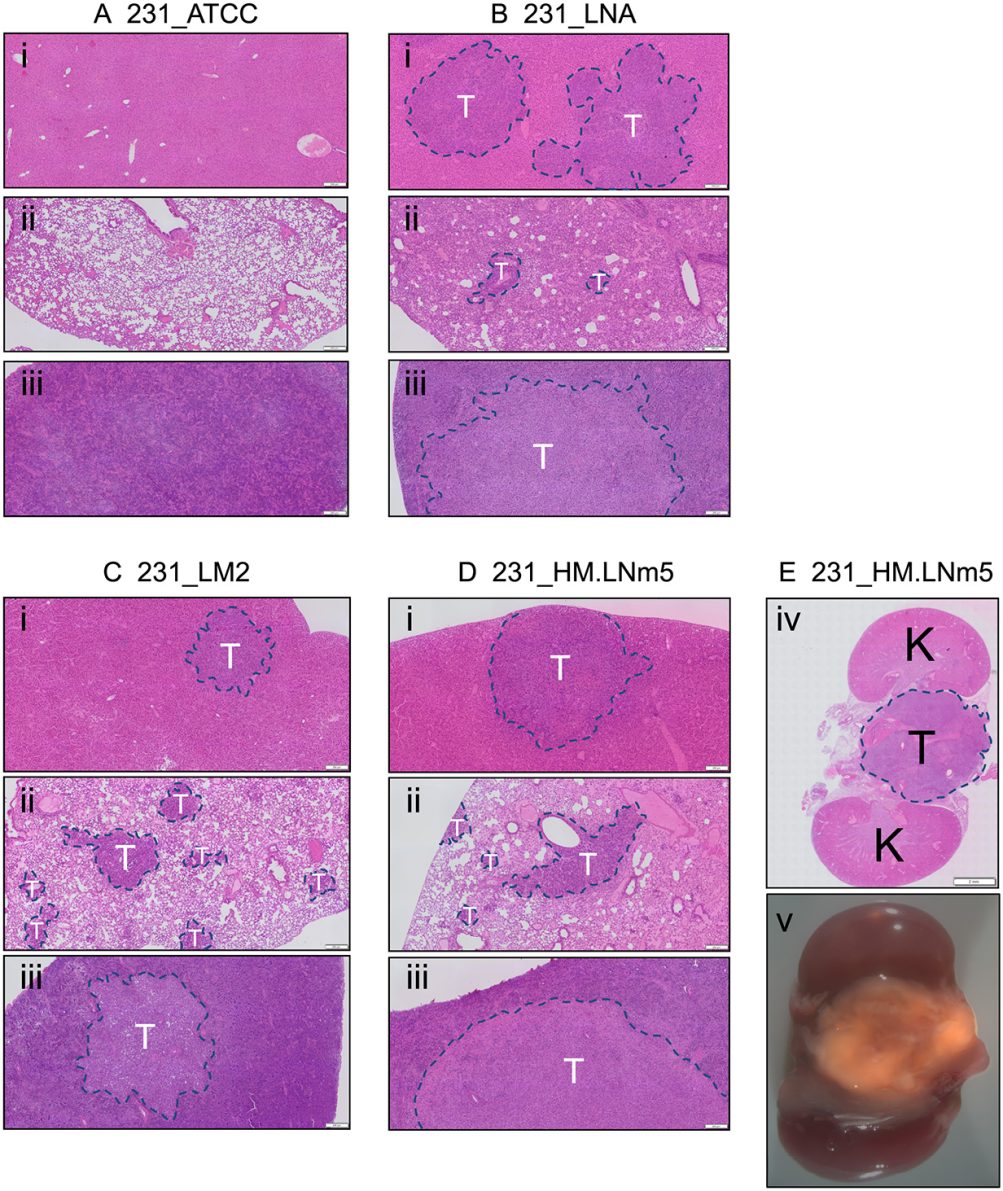
**Spontaneous metastasis of MDA-MB-231 variants to distant organs.** Sections (3 µm) were cut from representative FFPE-fixed secondary organs obtained 22 days after primary tumour resection. H&E-stained slides were scanned using an Olympus VS120 instrument and images generated using OlyVIA software (Olympus). (A) 231_ATCC. (B) 231_LNA. (C) 231_LM2. (D,E) 231_HM.LNm5. (i) Liver, (ii) lung, (iii) spleen, (iv) metastatic deposit on the spine between the kidneys, (v) merged brightfield and fluorescent (tdTomato) images of a metastatic deposit on the spine between the kidneys (7× magnification). Metastatic lesions are indicated with a dashed blue line. T, region of tumour; K, kidney. Scale bars: (i-iii) 200 µm, (iv) 2* *mm.

The relative abilities of the four tumour lines to metastasise spontaneously *in vivo* were compared side-by-side in a surgical resection model whereby single orthotopic mammary tumours were removed at a similar size (Fig. 1A, Figs S1 and S2). NOD.Cg-*Prkdc^scid^ Il2rg^tm1Wjl^*/SzJ (NSG) immunodeficient mice were used because they were previously shown to support increased metastasis of breast cancer lines (Iorns et al., 2012; Puchalapalli et al., 2016). Whole animal bioluminescence imaging (BLI) revealed that mice bearing 231_ATCC tumours failed to show evidence of either local or distant recurrence by 22 days after resection, whereas each of the three other tumour lines demonstrated both local recurrence and distant relapse in the abdominal and thoracic cavities (Fig. 1B). *Ex vivo* fluorescent imaging revealed metastatic deposits in lung, liver and spleen from animals inoculated with either 231_LNA, 231_LM2 or 231_HM.LNm5 cells (Fig. S3), which was associated with considerable whole-organ hypertrophy of liver and spleen (data not shown). No metastatic lesions were found in secondary organs of mice bearing 231_ATCC tumours (Fig. S3). These findings were confirmed and extended by histological analyses of lung, liver and spleen from tumour-bearing mice (Fig. 2). In addition to soft-tissue metastasis, 231_HM.LNm5 tumours also disseminated to spine in a minority of individuals (Fig. 2E). We also observed significant differences in the incidence of ipsilateral lymph node metastasis among the different models (Le et al., 2016). Metastasis to the draining lymph node in the axilla was observed in the 231_LM2 and 231_HM.LNm5 models but not in the 231_ATCC or 231_LNA lines (Fig. S4). Altogether, this extensive *in vivo* assessment demonstrates that the 231_ATCC model is incapable of spontaneous metastasis in NSG mice, whereas 231_LNA has an intermediate metastatic capacity, and the 231_LM2 and 231_HM.LNm5 tumours are highly metastatic.

**Fig. 3.**
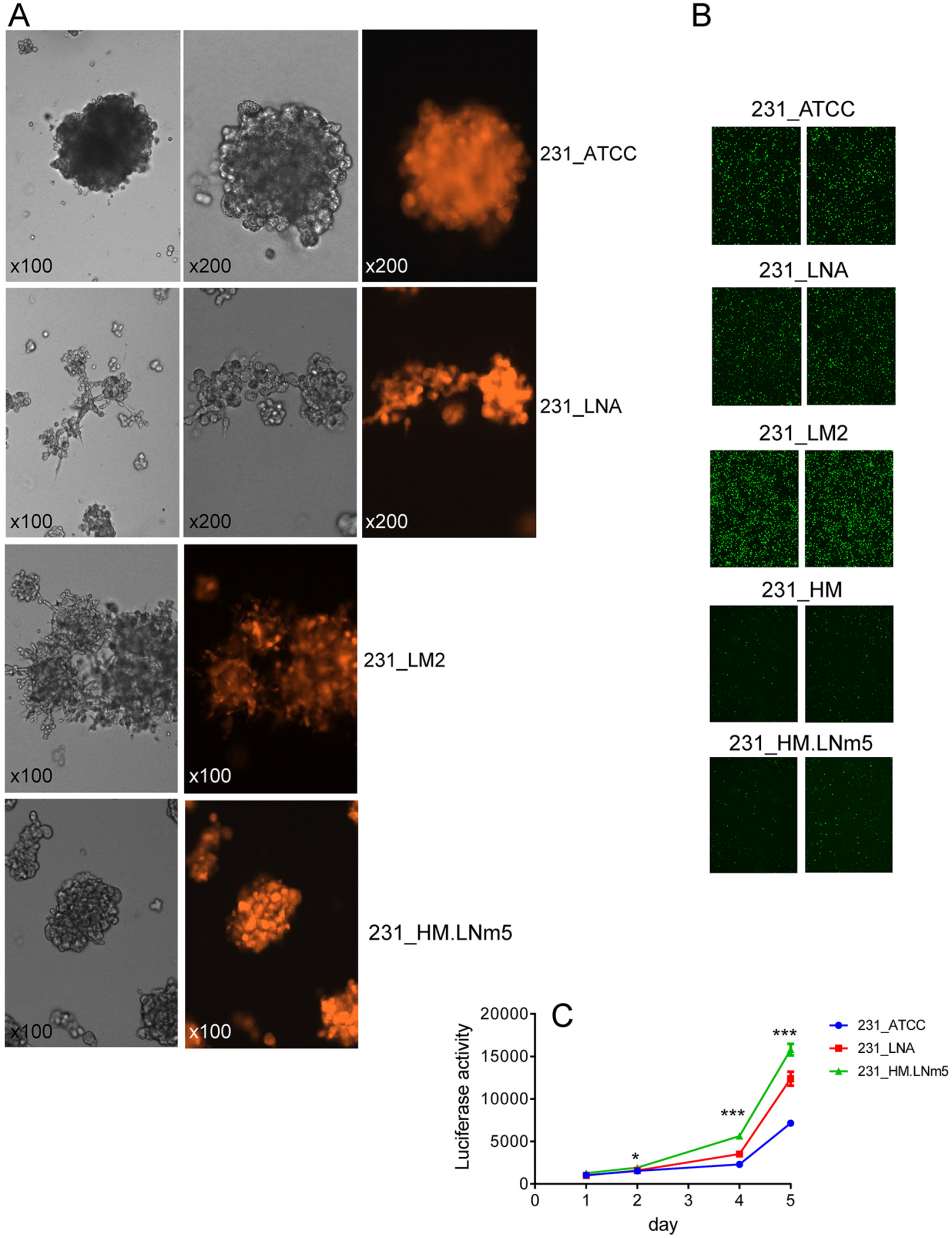
***In vitro* phenotypes of different MDA-MB-231 variants.** Proliferation rate, rather than an invasive growth pattern, is correlated with metastatic ability. (A) Images of cells cultured in 3D. The indicated cell lines were seeded on top of a 50% Cultrex matrix and images captured at the indicated magnifications after 5 days. Brightfield (greyscale) and matched tdTomato fluorescent images are shown. 231_ATCC and 231_HM.LNm5 lines formed non-invasive spheroids, whereas 231_LNA and 231_LM2 formed loosely connected invasive clusters. (B) *In vitro* motility of MDA-MB-231 variants. Transwell migration assays using 10% FBS as a chemoattractant (*n*=3 per line) were completed for the indicated cell lines. Parental MDA-MB-231HM (231_HM) cells were also included for comparison. Two representative fields (×100 magnification) from two different Transwells per cell line are displayed. (C) 3D *in vitro* proliferation (on 50% Cultrex) was measured over 5 days using the Cell Titer Glo assay (Promega) for the indicated cell lines. Mean±s.e.m. (*n*=4) is presented. *P<0.05. ***P<0.001 by Student's *t*-test.

231_ATCC was the slowest proliferating line, both *in vivo* (Fig. 1A) and in three-dimensional (3D) culture *in vitro* (Fig. 3C). 231_LNA and 231_LM2 formed loosely adherent invasive structures when cultured on a basement membrane gel *in vitro* (Fig. 3A), as expected for cancer cells with metastasising capability. However, despite the rapid proliferation and aggressive metastatic phenotype of 231_HM.LNm5 cells *in vivo*, they unexpectedly formed non-invasive near-spherical clusters in 3D culture, a phenotype that closely resembled the morphology of non-metastatic 231_ATCC cells (Fig. 3A). Both parental 231_HM cells and the 231_HM.LNm5 line showed substantially reduced *in vitro* migration towards serum than the other three lines (Fig. 3B). Indeed, we showed recently that 231_HM.LNm5 cells are less motile than the 231_ATCC cells *in vitro* (Fietz et al., 2017).

### Analysis of DNA content and DNA copy number in MDA-MB-231 variants

**Fig. 4.**
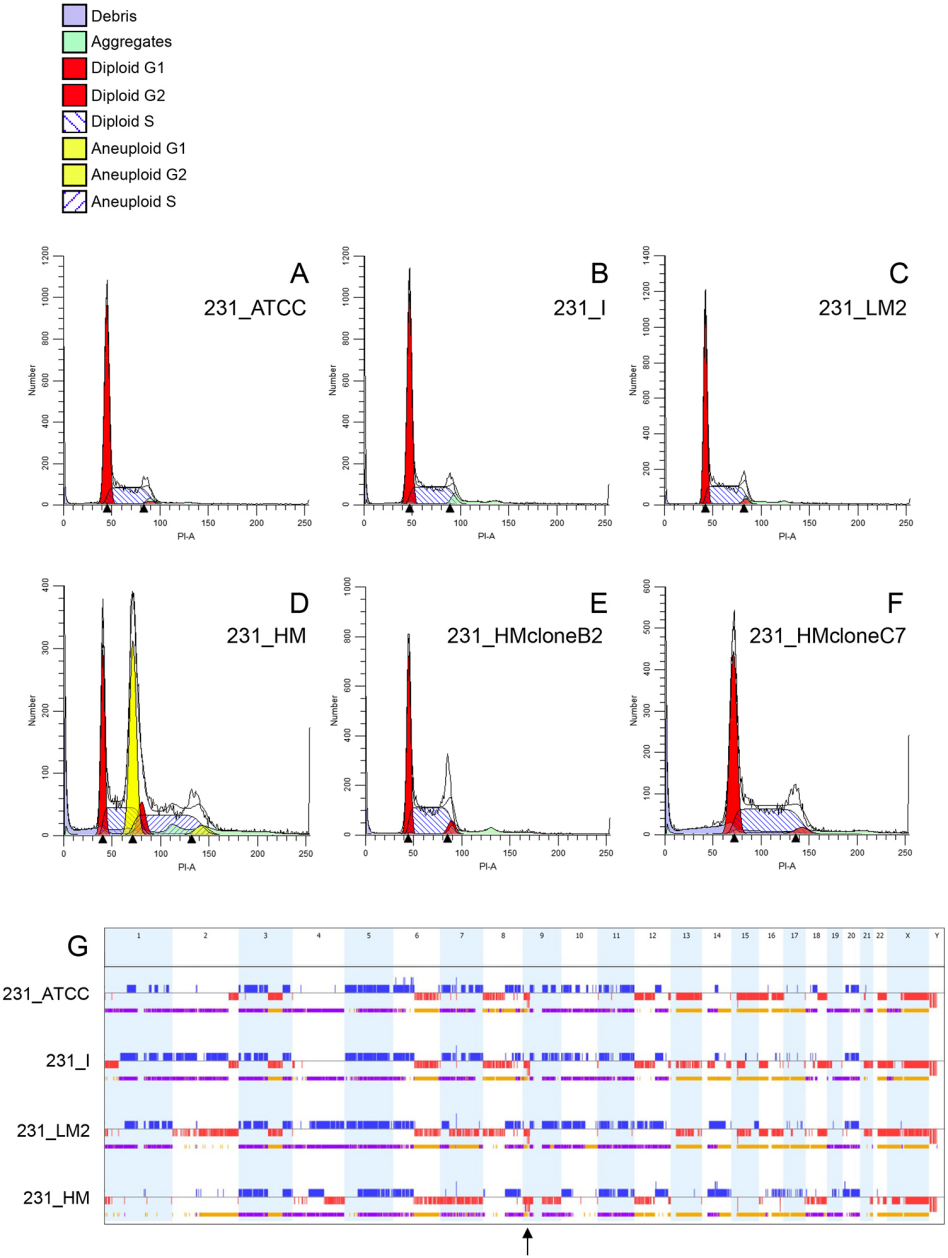
**Cell cycle and SNP array analysis of four MDA-MB-231 cell line variants.** (A-F) The indicated MDA-MB-231-derived cell lines were analysed for cell cycle distribution by flow cytometry. 231_HMcloneB2 (E) and 231_HMcloneC7 (F) are two different clonal daughter lines derived from parental 231_HM cells (D). (G) Genome-wide patterns of DNA copy number gain (blue), loss (red), loss of heterozygosity (yellow) and allelic imbalance (purple) derived from SNP array analysis are depicted linearly for the four indicated cell lines. The chromosome number is indicated at the top. The location of the homozygous deletion at *9p21.3* that is shared by the four cell lines is indicated by an arrow.

MDA-MB-231 cells are near triploid and display trisomy of most autosomes (Satya-Prakash et al., 1981). To assess potential heterogeneity within each MDA-MB-231-derived population, cell cycle analysis was conducted on asynchronously growing untagged versions of the four lines by flow cytometry. 231_ATCC, 231_I (from which 231_LNA was isolated) and 231_LM2 showed nearly identical DNA content and cell cycle distributions, with each suggestive of homogeneous or near-homogeneous populations (Fig. 4A-C). In contrast, parental 231_HM cells exhibited a cell cycle distribution indicative of two distinct cell populations (Fig. 4D). One population had an identical DNA content to the other three lines (representing 42% of the total), whereas an ‘aneuploid’ fraction represented the majority (58%) of the population. The ‘aneuploid’ cells displayed a 1.78-fold increase in total DNA content compared to the less abundant fraction. This is consistent with duplication of an entire diploid genome within near-triploid parental MDA-MB-231 cells, thus yielding a probable near-quintaploid daughter line. To explore this further, clonal populations of parental 231_HM cells were generated by limiting dilution and two daughter lines were characterised. 231_HMcloneB2 displayed a cell cycle distribution identical to that of the minor 231_HM fraction and to the other three cell lines (Fig. 4E). Conversely, 231_HMcloneC7 showed a distribution consistent with a homogenous population of the major near-quintaploid 231_HM population (Fig. 4F). These findings demonstrate that each of the two significant 231_HM populations can exist independently of one another. Both 231_HMcloneB2 and 231_HMcloneC7 lines were spontaneously metastatic to lung, liver and spleen when analysed in an orthotopic resection model *in vivo* (data not shown), suggesting that aggressive metastatic capacity is not specifically associated with DNA ploidy in the 231_HM model.

To define DNA copy number variations (CNVs) in the four MDA-MB-231-derived lines, genomic DNA from untagged versions of the cell lines (231_ATCC, 231_I, 231_LM2, 231_HM) cultured *in vitro* was applied to SNP arrays (Fig. 4G, Fig. S5). Each line showed a unique pattern of DNA copy number alteration, although common regions of gain/loss and allelic imbalance were also present, including shared homozygous deletion of the *CDKN2A* (encoding p16/INK4A and p14ARF)/*CDKN2B* (encoding p15/INK4B)/*MTAP* locus at *9p21.3* (Fig. 4G, Fig. S5). High level (CNV log_2_ ratio≥1) DNA amplification was not found in any line (Fig. 4G, Fig. S5). Through analysis of both shared and unique DNA copy number gains/losses across the genome (Table S2), the genetic divergence of each line from 231_ATCC was estimated. 231_LNA was most similar to 231_ATCC (15.2% difference), followed by 231_LM2 (25.8%), with 231_HM being the most divergent (66.1%). However, the latter result should be interpreted with caution since the 231_HM SNP array represents the merging of two different aneuploid populations.

### Differential gene expression among MDA-MB-231 variants

**Fig. 5.**
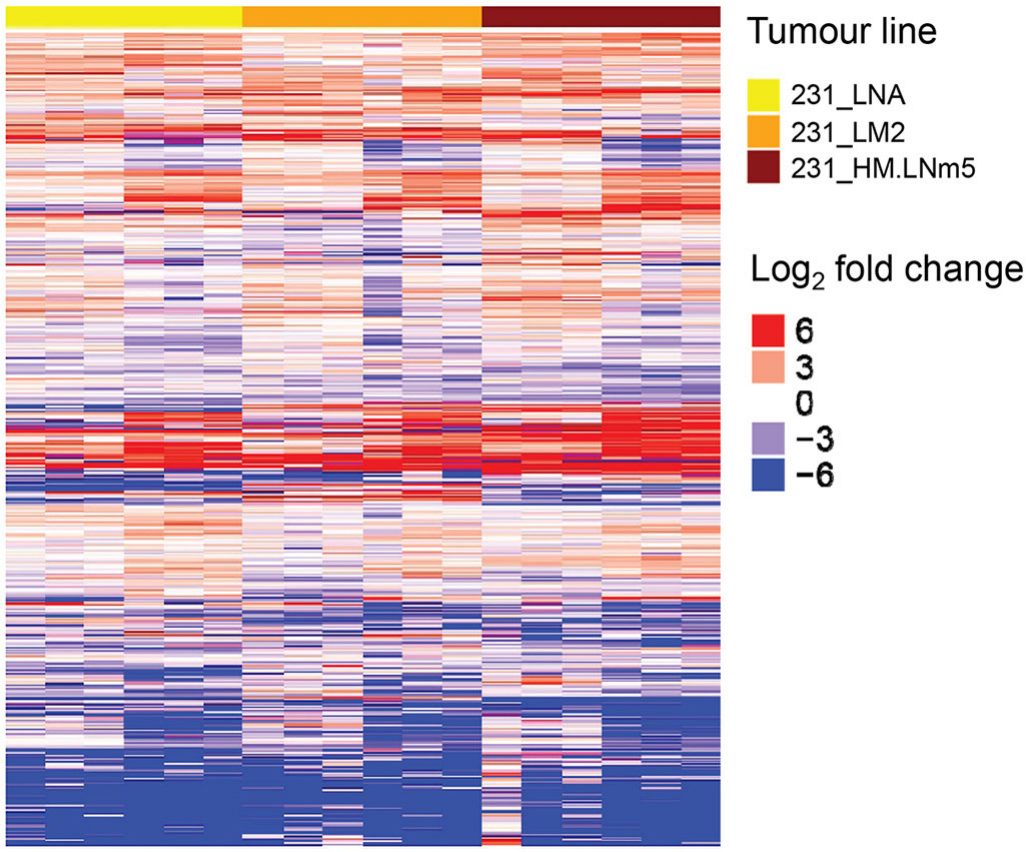
**Heat map of tumour cell gene expression patterns among MDA-MB-231 mammary xenografts.** Each row represents a single gene. Rows were clustered by similarity (Euclidian distance). Only genes with both a ≥twofold change (log_2_ fold change ≤−1 or ≥1) in expression level compared to non-metastatic 231_ATCC tumour cells (*n*=2) and a mean sequencing transcript count of ≥30 are shown. 231_LNA (*n*=6), 231_LM2 (*n*=6), 231_HM.LNm5 (*n*=6). The full dataset of differentially expressed genes is provided in Table S3.

To identify the factor(s) underlying the vastly different metastatic capacities of the four congenic models, resected primary tumours were disaggregated, total RNA recovered from isolated cancer cells (Fig. S1) and digital RNA sequencing (RNA-Seq) conducted (Harrison et al., 2015). Consistent patterns of differential gene expression were found among the four MDA-MB-231 variants (Fig. 5). Gene set enrichment analysis (GSEA) was carried out on the RNA-Seq data to ascertain cellular processes that were deregulated in all three metastatic lines (231_LNA, 231_LM2, 231_HM.LNm5) compared to the non-metastatic 231_ATCC line (Subramanian et al., 2005), and therefore might drive a metastatic phenotype. Remarkably, of the ten most significantly upregulated gene sets in metastatic cells, nine were related to RNA splicing and metabolism, or to ribosome biogenesis (Table 1, Fig. S6). To extend these findings, The Cancer Genome Atlas (TCGA) breast cancer dataset was then interrogated (Koboldt et al., 2012). Comparison of small tumours from stage I patients to primary neoplasms from patients with stage II-IV disease revealed that each of the nine data sets concerning RNA metabolism were also significantly upregulated in tumours from later stage patients and hence could be causally involved in the progression of human breast cancer (Table 1). Interestingly, significant downregulation of genes involved in mammary gland development and morphogenesis was also found (Table 1), perhaps indicating that the acquisition of metastatic competency in MDA-MB-231 variants was associated with de-differentiation. One possible explanation for the GSEA results is that increased expression of ribosome biogenesis genes was a reflection of the enhanced requirement for protein synthesis in the faster-proliferating metastatic cells compared to 231_ATCC cells (Orsolic et al., 2016; van Riggelen et al., 2010). However, no evidence was found for increased expression of any gene sets involved in epithelial cell proliferation in the metastatic lines (Table 1). One process that can influence acquisition of metastatic capability in breast cancer is epithelial-to-mesenchymal transition (EMT), whereby a more mesenchymal phenotype can endow cancer cells with the ability to degrade basement membrane and invade into the surrounding tissue (Chaffer et al., 2016; Taube et al., 2010). To test for dysregulation of genes relevant to EMT, GSEA was carried out using ‘core EMT’ genes either up- or downregulated after induction of EMT in human mammary epithelial cells (Taube et al., 2010). The set of epithelial genes downregulated upon induction of EMT were significantly repressed in the three metastatic models compared to 231_ATCC (Table 1). This suggested that, despite MDA-MB-231 possessing a mesenchymal or ‘basal B’ phenotype (Lehmann et al., 2011; Neve et al., 2006), further reductions in epithelial gene expression levels are associated with acquisition of metastatic potential in MDA-MB-231-derived metastatic variants. Significant changes in mesenchymal genes upregulated upon EMT induction were not found (data not shown). To further probe this aspect, the expression and distribution of a key epithelial marker (E-cadherin) and a key mesenchymal marker (vimentin) were examined. E-cadherin mRNA was not detected in MDA-MB-231-derived tumour cells by RNA-Seq and E-cadherin protein was not present at cell-cell junctions in any of the four lines when evaluated *in vitro* or *in vivo* (Fig. S7B). Vimentin mRNA was abundant in the tumour cells of each model (data not shown), but was not differentially expressed (Fig. S7A). A filamentous cytoplasmic staining pattern for vimentin was present in each of the four cell lines *in vitro* and strong cytoplasmic staining was revealed in each line *in vivo* (Fig. S7C). All four tumour models stained weakly positive when a pan-cytokeratin (CK) antibody (recognising CK1, CK5, CK6 and CK8) was applied (Fig. S8). Furthermore, no differences in either E-cadherin or vimentin staining intensity or distribution were observed in the two clonal 231_HM daughter lines, 231_HMcloneC7 and 231_HMcloneB2 (Fig. S9). Together, these data show that each of the four models possess a strong mesenchymal phenotype.

**Table 1.**
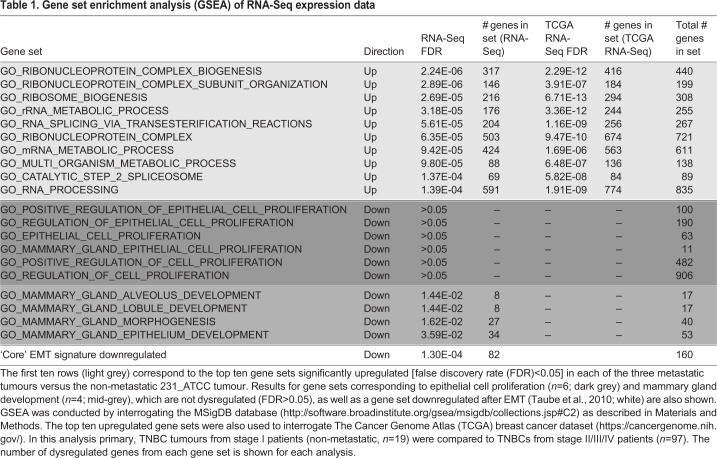
**Gene set enrichment analysis (GSEA) of RNA-Seq expression data**

Dysregulation of individual genes across the four models was also observed and some of these could be grouped into broad categories or families (Table 2). We focused on two patterns of altered gene expression (Nguyen et al., 2009). We first examined transcripts whose expression was deregulated in all metastatic models versus non-metastatic 231_ATCC primary tumour cells. In this context, upregulated genes were designated as metastasis initiation genes and, conversely, downregulated genes were labelled as metastasis suppression genes (Tables S3-S4, Fig. S10). In a similar vein, mRNAs with augmented expression specifically in highly metastatic 231_HM.LNm5 cells compared with the other three models were labelled as metastasis virulence genes, whereas the corresponding downregulated genes were categorised as metastasis avirulence genes (Tables S3-S4, Fig. S10).

**Table 2.**
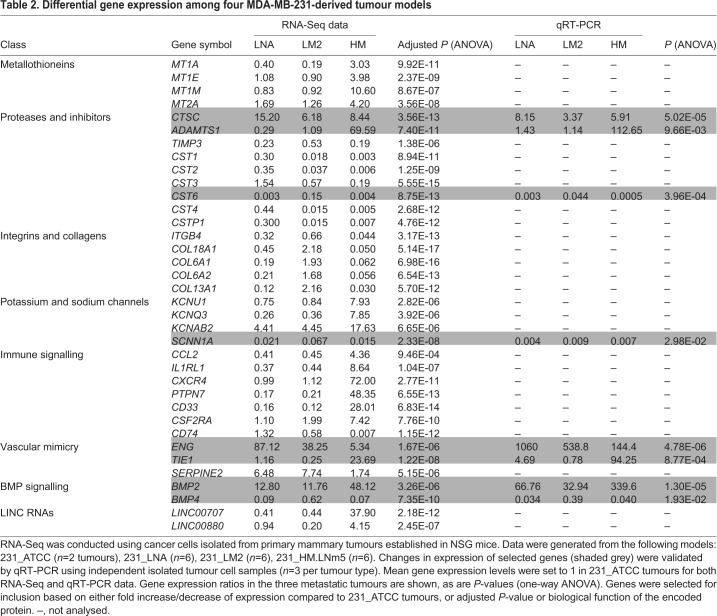
**Differential gene expression among four MDA-MB-231-derived tumour models**

Classes of molecules with confirmed roles in tumour progression were dysregulated (Table 2). The cysteine proteinase cathepsin C [*CTSC* (Ruffell et al., 2013)] was upregulated in all three metastatic models, whereas several of the type 2 cystatin genes, inhibitors of cysteine proteinases, were downregulated in each (Ai et al., 2006; Cox, 2009). The bone morphogenetic proteins BMP2 and BMP4 were dysregulated. *BMP2*, reported to promote invasion and progression of MCF-7 breast cancer cells (Clement et al., 2005), was substantially elevated in all three metastatic lines, whereas the breast cancer metastasis suppressor gene *BMP4* (Cao et al., 2014) was suppressed in each of the three variants (Table 2). Expression of *BMPRIA*, which encodes the preferred receptor for both BMP2 and BMP4, was unchanged (data not shown). Interestingly, altered expression of gene clusters was also noted. Metallothioneins are involved in the cellular metabolism of metal ions (Dziegiel et al., 2016). Four genes from the metallothionein cluster on human chromosome 16q13 (*MT1A*, *MT1E*, *MT1M*, *MT2A*) were overexpressed in 231_HM.LNm5 tumours only (Table 2), thus placing them in the metastatic virulence category (Table S4). Similarly, three genes from the collagen locus on 21q22 (*COL6A1*, *COL6A2*, *COL18A1*) were specifically repressed in 231_HM.LNm5. However, the type 2 cystatins (*CST1*, *CST2*, *CST4*) and the cystatin pseudogene *CSTP1*, all encoded by a gene cluster on 20p11, were downregulated in the three metastatic models compared to non-metastatic 231_ATCC. Additionally, *CST6*, a related type 2 cystatin gene located on a different chromosome, was strongly repressed in each of the three metastatic lines (Table 2).

Shao and colleagues previously reported increased angiogenesis in MDA-MB-231HM primary xenografts compared to tumours formed by the parental MDA-MB-231 line, as determined by staining for microvessel density and microangiography (Jin et al., 2012), and our results concur with this (data not shown). To determine whether hypoxia might play a role in augmented neo-angiogenesis in the 231_HM.LNm5 primary tumours, the RNA-Seq data was analysed for hypoxia-induced pro-angiogenic factors. Surprisingly, the average expression level of the key angiogenesis-promoting gene, *VEGFA* (Ferrara, 2002), was lowest in the 231_HM.LNm5 tumour cells, and significantly lower than in non-metastatic 231_ATCC cells (Fig. S11Ai). Likewise, average levels of hypoxia-responsive carbonic anhydrase 9 (*CA9*) were also lowest in 231_HM.LNm5 (Fig. S11Aii). *In vitro* experiments revealed a modest elevation of *VEGFA* mRNA expression in 231_ATCC cells following exposure of cells to 1% oxygen for 6 h or 24 h, and this induction was blunted in 231_HM.LNm5 cells (Fig. S11B). This suggested that other factors in addition to VEGFA may promote vigorous angiogenesis in 231_HM.LNm5 primary xenografts.

Another process that increases blood supply to the tumour is vascular mimicry, whereby cancer cells transdifferentiate into endothelial-like cells to form blood-carrying tubular structures that can relieve hypoxia and also promote metastasis in mouse models (Seftor et al., 2012; Wagenblast et al., 2015). Three genes that contribute to vascular mimicry, endoglin (*ENG*; a component of the transforming growth factor beta receptor complex on endothelial cells), *TIE1* (encoding a key angiogenic receptor) and the vascular network-promoting anti-coagulant *SERPINE2* (Wagenblast et al., 2015), were all upregulated in metastatic cells (Table 2). Of note, endoglin is able to bind BMP-2 by interacting with the ligand-binding BMP type I receptors ALK3 (BMPRIA) and ALK6 (BMPRIB) (Barbara et al., 1999), but whether ectopically upregulated BMP2 and ENG form a functional receptor complex in metastatic MDA-MB-231 variants remains to be elucidated. Thus, vasculogenic mimicry is a possible phenomenon promoting tumour progression, but further evaluation is required to confirm its presence and potential role in haematogenous metastasis of MDA-MB-231 variants. Finally, several genes encoding ion channels were dysregulated among the four tumours (Table 2). Expression of the voltage-gated potassium channels KCNU1, KCNQ3 and KCNAB2 was increased in highly metastatic lines, whereas *SCNN1A*, encoding the alpha subunit of the major epithelial sodium channel (non-voltage-gated) and regulated by glucocorticoids (Fietz et al., 2017), was downregulated in all three metastatic tumours and is a candidate metastasis suppressor gene (Table 2). The C-C motif chemokine ligand-2 (CCL2) promotes breast cancer metastasis through engagement with its receptor CCR2 on macrophages (Kitamura et al., 2015). *CCL2* mRNA levels were at least fourfold higher in 231_HM.LNm5 primary tumour cells compared to the three other lines (Table 2). In addition, secreted CCL2 protein was undetectable in 231_ATCC, 231_LNA and 231_LM2 3D cultures *in vitro* but was robustly expressed by 231_HM.LNm5 cells (data not shown). Interestingly, work by Kang and Lu showed that ectopic expression of CCL2 in 231_LM2 cells enhanced experimental metastasis to lung via recruitment of tumour-promoting lung macrophages that display its cognate receptor CCR2 (Lu and Kang, 2009). Indeed, targeting the CCL2-CCR2 axis is being evaluated for therapy of breast cancer, although recent findings suggest that modulation of this pathway may result in deleterious unintended consequences in patients (Bonapace et al., 2014).

### Gene methylation is associated with acquisition of aggressive metastatic capacity

**Fig. 6.**
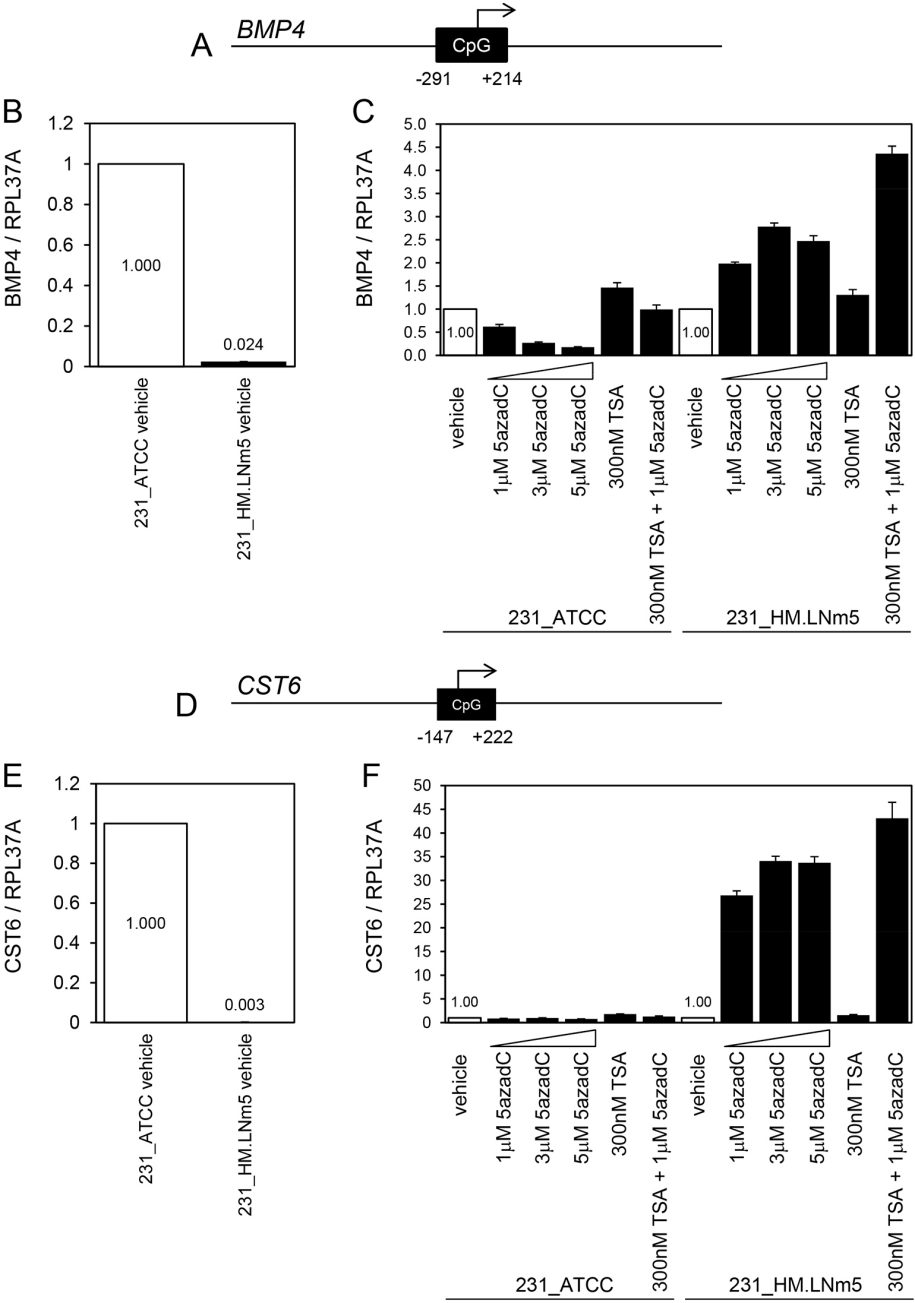
**Analysis of *BMP4* and *CST6* mRNA levels following treatment with demethylating agent and histone deacetylase inhibitor.** (A) A CpG island (506 bp) spans the transcription start site of the human *BMP4* gene. (B) Relative *BMP4* mRNA levels in vehicle (DMSO)-treated 231_ATCC and 231_HM.LNm5 cells by TaqMan qRT-PCR. Expression in 231_ATCC was set to 1. Mean±s.d. (*n*=3). (C) TaqMan qRT-PCR analysis of *BMP4* mRNA expression levels in 5-Aza-2′-deoxycytidine (5azadC)- and Trichostatin A (TSA)-treated 231_ATCC and 231_HM.LNm5 cells. Expression in vehicle-treated cells was set to 1. Mean±s.d. (*n*=3). (D) A CpG island (370 bp) spans the transcription start site of the human *CST6* gene. (E) Relative *CST6* mRNA levels in vehicle (DMSO)-treated 231_ATCC and 231_HM.LNm5 cells by TaqMan qRT-PCR. Expression in 231_ATCC was set to 1. Mean±s.d. (*n*=3). (F) TaqMan qRT-PCR analysis of *CST6* mRNA expression levels in 5azadC- and TSA-treated 231_ATCC and 231_HM.LNm5 cells. Expression in vehicle-treated cells was set to 1. Mean±s.d. (*n*=3).

Since robust alterations in gene expression were evident across the four tumour models from the RNA-Seq data, a preliminary investigation into the mechanisms underlying these differences was conducted. Gene CNVs are unlikely to underlie the substantial differences in gene expression observed because high-level amplifications and homozygous deletions were absent from all lines with the exception of deletion at *9p21.3* (*CDKN2A/B*), which was observed in all four cell lines (Fig. 4G). Changes in promoter methylation are associated with gain of metastatic potential in breast cancer (Fang et al., 2011), so we investigated whether DNA methylation could be an alternative explanation for the marked suppression of selected genes in 231_HM.LNm5 tumour cells by using DNA demethylating agents and histone deacetylase inhibitors (Cameron et al., 1999). Both *BMP4* and *CST6* are metastasis suppression genes downregulated in 231_HM.LNm5 tumour cells relative to non-metastatic 231_ATCC tumour cells (Table 2), and both contain CpG islands spanning their transcription start sites in their 5′ proximal regions (Fig. 6A,D). Suppression of *BMP4* (Fig. 6B) and *CST6* (Fig. 6E) levels in 231_HM.LNm5 cells relative to 231_ATCC cells was retained in 2D culture, suggesting that the mechanism(s) underlying downregulation are cancer cell intrinsic. Treatment with the demethylating agent 5-Aza-2′-deoxycytidine (5azadC) resulted in a modest two- to threefold induction of *BMP4* mRNA in 231_HM.LNm5 cells but no induction in 231_ATCC cells (Fig. 6C). The histone deacetylase (HDAC) inhibitor Trichostatin A (TSA) produced only a negligible increase (1.3- to 1.5-fold) in *BMP4* mRNA levels in both cell lines, showing that histone acetylation alone is not responsible for *BMP4* silencing in 231_HM.LNm5 cells. Co-administration of TSA and 5azadC led to a further increase (4.4-fold) in BMP4 expression specifically in the 231_HM.LNm5 cells. Treatment with 5azadC resulted in a marked 27- to 34-fold increase in *CST6* mRNA levels in 231_HM.LNm5 cells but no increase in 231_ATCC cells (Fig. 6F). As found for BMP4, TSA treatment enhanced CST6 expression only modestly (1.6- to 1.8-fold) in both lines. TSA and 5azadC co-treatment elevated *CST6* mRNA 43-fold in 231_HM.LNm5 cells but again had no effect in 231_ATCC cells.

Together, these data indicate that the key CpG islands of *BMP4* and *CST6* spanning their transcription start sites are not methylated in 231_ATCC cells, and also suggest potential low-level methylation of *BMP4* and robust methylation of *CST6* in 231_HM.LNm5 cells, although direct assessment of CpG island methylation is required to answer this conclusively. Work by others showed methylation of *CST6* to be associated with worse patient prognosis in breast cancer (Kioulafa et al., 2009), whereas the encoded protein (cystatin M) inhibited breast cancer cell proliferation, motility and metastasis (Zhang et al., 2004). *BMP4* was also shown to be methylated in breast cancer (Hartmann et al., 2009). Interestingly, CpG island methylation was also associated with EMT in a human breast cancer cell line model (Carmona et al., 2014), and could therefore contribute to the downregulation of epithelial genes seen in the three metastatic MDA-MB-231 variants compared with 231_ATCC (Table 1).

## DISCUSSION

### Associations among proliferation, motility, angiogenesis and spontaneous metastasis in MDA-MB-231 variants

Characterisation of mouse models of TNBC has improved our understanding of the cellular, molecular and physiological processes underlying multi-step metastasis; however, additional models with spontaneous metastatic capacity that are amenable to both gene modification and preclinical testing of experimental therapeutics would be beneficial.

Simultaneous assessment of the four MDA-MB-231-derived lines with different spontaneous metastatic potential showed that growth rate is correlated with metastatic ability in MDA-MB-231 variants, whereas invasive growth in 3D culture and *in vitro* migration are not, which concurs with published data (Fietz et al., 2017). This suggests that the ability of TNBC cells to invade through basement membrane is not the sole determinant of metastatic proclivity *in vivo* (Narod and Sopik, 2018), but rather that primary tumour proliferation rate may be better correlated with intrinsic metastatic potential, as suggested by others based on analysis of MDA-MB-231 variants (Minn et al., 2007).

Despite the increased angiogenesis found in 231_HM.LNm5 primary tumours compared to the other three lines (Jin et al., 2012 and data not shown), average levels of the central hypoxia-induced pro-angiogenic factor VEGFA were unexpectedly lowest in 231_HM.LNm5 and the induction of *VEGFA* mRNA by hypoxia was blunted in 231_HM.LNm5 cells compared to 231_ATCC cells *in vitro*. Along with low levels of CA9, this suggests that exponentially growing 231_HM.LNm5 tumours may experience less extreme hypoxia than the other tumour types and also that other factors may compensate for low VEGFA to promote angiogenesis. IL-8 protein was strongly expressed by 231_HM.LNm5 cells *in vitro* (data not shown) and was shown previously to compensate for reduced VEGFA levels to sustain angiogenesis in colorectal cancer xenografts (Mizukami et al., 2005). In addition, CCL2, which was upregulated in 231_HM.LNm5 tumour cells, can indirectly promote tumour angiogenesis via recruitment of macrophages (Low-Marchelli et al., 2013). Therefore, the 231_HM.LNm5 model should prove useful for the study of angiogenesis in primary breast cancers and its relationship to haematogenous metastasis.

### Differential gene expression in primary tumour cells from MDA-MB-231 orthotopic xenografts

Unexpectedly, GSEA showed that several gene networks related to ribosome biogenesis and RNA metabolism were upregulated both in metastatic MDA-MB-231 cells as well as in whole human primary TNBCs corresponding to a more advanced stage. Although the vast majority of genes in each gene network were upregulated (Table 1), the magnitude of the change for individual genes in the three metastatic lines was generally subtle (data not shown), suggesting coordinate regulation at a systems level. Activity of these pathways is often correlated with cell proliferation in order to ensure enough translational output for cell division (Orsolic et al., 2016; van Riggelen et al., 2010). However, while each of the three metastatic MDA-MB-231 variants grew faster than parental 231_ATCC cells both *in vitro* and *in vivo*, gene sets related to the positive regulation of epithelial cell proliferation were not augmented in metastatic cells *in vivo*. Enhanced ribosome biogenesis and RNA metabolic pathways could also play a direct role in metastatic dissemination independently of effects on cell proliferation. Pursuit of this possibility will require consideration of the activity of the key regulator of translation, mTORC1, and its downstream effectors p70S6K and 4E-BP1 (Steelman et al., 2016).

Genome-wide expression profiling of the primary tumour cells isolated from orthotopic xenografts enabled the delineation of both previously described and novel genes and gene families associated with pro- or anti-metastatic functions. The ease with which these breast tumour lines can be propagated both *in vitro* and *in vivo* and gene expression altered in a stable manner will allow the elucidation of the precise function(s) of dysregulated genes in the metastatic cascade, particularly for those genes likely to operate in a tumour cell autonomous mode. For example, the potential metastasis suppressor activities of *CST1*, *CST2*, *CST4*, *CST6*, *BMP4* and *SCNN1* could be individually tested by coupling silencing of endogenous expression in non-metastatic 231_ATCC cells with stable restoration of expression in the metastatic 231_LM2 and 231_HM.LNm5 lines. Conversely, the putative pro-metastatic activities of *ADAMTS1*, *CTSC*, *BMP2*, *ENG* and *TIE1* could be tested by ectopic over-expression in 231_ATCC cells paired with stable depletion in the 231_HM.LNm5 line. In addition to the assessment of gene function, the lines should be useful for the testing of experimental therapeutics, particularly specific small molecule inhibitors of the proteinases ADAMTS1 and CTSC and of the transmembrane tyrosine kinase TIE1.

## MATERIALS AND METHODS

### Cell lines and cell culture

The MDA-MB-231 human breast adenocarcinoma cell line was purchased from ATCC (Cailleau et al., 1974) and designated 231_ATCC. The MDA-MB-231_LM2 variant (231_LM2) (also designated clone 4175) was a kind gift from Joan Massague (Memorial Sloan Kettering Cancer Center, USA) (Minn et al., 2005a). The MDA-MB-231_LNA (231_LNA) line was isolated from a spontaneous lymph node metastasis that arose in a mouse inoculated orthotopically with late-passage MDA-MB-231_I cells (Fig. S1, Table S1; Johnstone et al., 2008). MDA-MB-231HM cells (Chang et al., 2007, 2008) were kindly provided by Zhi Min Shao and Zhou Luo Ou (Breast Cancer Institute, Fudan University, Shanghai, China). The MDA-MB-231HM.LNm5 (231_HM.LNm5) variant was derived as described (Fietz et al., 2017). To obtain clonal sublines of parental MDA-MB-231HM cells, cells were transduced with the pFB_neo_Fluc retroviral vector (a kind gift from Hiroshi Nakagawa, University of Pennsylvania, PA) and selected with G418 (1000 µg/ml) for 2 weeks, followed by seeding of single viable cells using flow cytometry (FACSDiva, Beckton Dickinson, Franklin Lakes, NJ, USA). Luciferase activity positive colonies were expanded and two clones (231_HMcloneC7 and 231_HMcloneB2) were selected for further analysis. All lines were maintained in Phenol-Red-containing Dulbecco's modified Eagle medium (DMEM; Thermo Fisher Scientific, Scoresby, Victoria, Australia) supplemented with 10% (v/v) heat-inactivated foetal bovine serum (FBS; Thermo Fisher Scientific), 15 mM HEPES buffer, 2 mM L-glutamine, 1% (v/v) non-essential amino acids, 5% (v/v) sodium pyruvate, penicillin (100 IU/ml) and streptomycin (100 µg/ml). Cells were maintained at 37°C in 5% CO_2_ (v/v) in air and sub-cultured every 4-5 days. For 3D culture, cells were seeded onto a solid basement membrane gel (50% Cultrex in full medium; Trevigen, Gaithersburg, MD, USA). MDA-MB-231-derived cell lines were verified for authenticity using the AmpFLSTR™ Identifiler™ PCR Amplification Kit (Thermo Fisher Scientific) at CellBank Australia (http://www.cellbankaustralia.com) and confirmed using the GenePrint^®^ 10 System (Promega Corporation, Alexandria, NSW, Australia) at the QIMR Berghofer Medical Research Institute, Australia. According to the International Cell Line Authentication Committee (ICLAC; http://iclac.org/), cell lines are considered authentic if >80% of measured alleles [evaluation value (EV)>0.8] matched those of the repository sample (Capes-Davis et al., 2013). Individual EVs for the parental versions of the cell lines were: 231_ATCC (1.00), 231_I (1.00), 231_LM2 (0.80), 231_HM (0.96).

### Treatment of cell lines with 5-Aza-2′-deoxycytidine and Trichostatin A

The DNA demethylating agent 5azadC and the histone deacetylase inhibitor TSA (Cayman Chemical, Ann Arbor, MI, USA) were dissolved in DMSO. Cells were treated with increasing doses of 5azadC or vehicle (DMSO) for 4 days, replacing the drug every 24 h. Cells were incubated with TSA alone for 24 h. For co-administration experiments, cells were first incubated with 5azadC (1 µM) for 3 days, followed by co-incubation with 5azadC (1 µM) and TSA (300 nM) for 24 h.

### Generation of reporter-gene-expressing cell lines

The fluorescent protein-tagged and luciferase-tagged cell lines used in the comparison of MDA-MB-231 variants are summarized in Table S1. Briefly, amphotropic retroviruses encoding reporter genes were generated using 293T-based Phoenix-Ampho packaging cells (Swift et al., 2001). Viral supernatants were filtered through 0.45 µm membranes and target cells transduced using the spin-infection method as previously described (Johnstone et al., 2008). Stably transduced cells were selected in the appropriate antibiotic followed by sorting for expression of fluorescent protein by flow cytometry (FACSDiva). Luciferase-expressing daughter lines co-expressing eGFP and/or tdTomato proteins (outlined in Table S1) were used in all *in vitro* and *in vivo* assays unless otherwise stated (Shaner et al., 2005). STR analysis was used to authenticate (EVs>0.80) all four reporter-gene-tagged MDA-MB-231 variants (data not shown).

### Cell cycle analysis by flow cytometry

Parental 231_ATCC, 231_I, 231_LM2 and 231_HM cells in exponential growth phase were trypsinised and 5×10^5^ cells fixed with cold 70% ethanol. Cells were washed twice with cold PBS and then incubated with RNaseA and propidium iodide (PI) in a total volume of 300 µl. At least 10,000 single cells were analysed by flow cytometry (FACSCanto, Beckton Dickinson, Franklin Lakes, NJ, USA). Flow cytometry data were analysed with ModFit LT v5.0 software (Verity Software House, Topsham, ME, USA) using the manual setting. All coefficient of variation (CV) values were <6.0%.

### Protein expression and distribution by immunofluorescence

Cells were cultured on 8-well plastic chamber slides (Nunc^®^ Lab-Tek^®^ Chamber Slide™ system, Sigma Aldrich), fixed with 4% paraformaldehyde for 30 min and permeabilised using 0.1% Triton X-100 for 5 min. Slides were blocked with 10% horse serum/1% bovine serum albumin (1 h at room temperature). Mouse anti-human E-cadherin monoclonal antibody (clone 36, 1:100 dilution, BD Transduction Labs) or mouse anti-human vimentin monoclonal antibody (clone V9, 1:500 dilution, eBioscience, Thermo Fisher Scientific) were used with an Alexa-Fluor-568-conjugated anti-mouse secondary antibody (Molecular Probes). Nuclei were visualised using DAPI (Sigma Aldrich) and images generated using a LSM780 inverted confocal microscope (Carl Zeiss, North Ryde, NSW, Australia) with a 10× objective and associated ZEN software (Zeiss).

### Protein expression and distribution by immunohistochemistry

Orthotopic xenografts were resected from mouse mammary glands and fixed in 10% neutral buffered formalin for 4-8 h. Heat-induced antigen retrieval was conducted for 15 min on 4 µm sections submerged in citrate buffer (pH 6.0). Sections were blocked with 10% horse serum/1% bovine serum albumin (1 h at room temperature) and then incubated overnight (4°C) in primary antibody. Mouse anti-human E-cadherin monoclonal antibody (clone 36, 1:100 dilution, BD Transduction Labs), mouse anti-human vimentin monoclonal antibody (clone V9, 1:500 dilution, eBioscience, Thermo Fisher Scientific), or mouse anti-human pan-cytokeratin monoclonal antibody (clone PCK-26 ascites, 1:300 dilution, Sigma Aldrich) were used. PCK-26 recognises an epitope on the type II cytokeratins 1, 5, 6 and 8. Sections were incubated with a horseradish peroxidase (HRP)-conjugated anti-mouse secondary antibody (Dako, Agilent Technologies, Mulgrave, Victoria, Australia) for 1 h at room temperature prior to timed incubation with the chromogen 3,3'-diaminobenzidine (Dako). Sections were counterstained with haematoxylin to visualise nuclei (blue) and slides were scanned using an Aperio Digital Pathology Slide Scanner (Leica Microsystems, Mount Waverley, Victoria, Australia). Images were generated using Aperio ImageScope software (Leica Microsystems).

### Isolation of total RNA from primary tumour cells

Resected primary tumours were disaggregated by digestion with collagenase I (Worthington Biochemical Corporation, Lakewood, NJ, USA) and bovine pancreatic DNase I (Roche Diagnostics, North Ryde, NSW, Australia) at 37°C and filtered through a series of sieves prior to sorting of viable tumour cells by flow cytometry (FACSDiva, Beckton Dickinson). Hydroxystilbamidine (Fluoro-Gold, Abcam, Melbourne, Victoria, Australia) was used to exclude non-viable cells. Total RNA was prepared from sorted cells using the Direct-zol RNA mini kit (Zymo Corporation, Irvine, CA, USA) incorporating on-column DNase I digestion in accordance with the manufacturer's instructions.

### Quantitative real-time RT-PCR (qRT-PCR)

First-strand cDNA was oligo dT-primed and synthesised from 5 µg total RNA with M-MLV reverse transcriptase in a 20 µl reaction volume (Promega). Two-step qPCR was completed (50-250 ng cDNA template/reaction) using either TaqMan gene expression assays (CTSC, ENG, TIE1, BMP2, BMP4, RPL37A) with Fast Universal PCR Master Mix, no AmpErase™ UNG (Thermo Fisher Scientific) or Fast SYBR™ Green Master Mix (50-250 ng cDNA template/reaction) and the following oligonucleotide primers (Integrated DNA Technologies, Singapore). ADAMTS1, F 5′-AGCCCAAGGTTGTAGATG-3′, R 5′-GCTTTTACACACTGTCCTTG-3′; CST6, F 5′-TCCGAGACACGCACATCATC-3′, R 5′-CCCATCTCCATCGTCAGGAA-3′; SCNN1A, F 5′-TACTGCTACTATAAGCTCCAG-3′, R 5′-TGTTGTTGACGGTGTAATTG-3′; RPL37A was used as an internal reference gene. Reactions (10 µl volume) were conducted for 50 cycles using either Step One Plus or ViiA 7 real-time PCR instruments (Thermo Fisher Scientific).

### Expression profiling by RNA-Seq

Total RNA integrity was checked using an RNA6000 Nano chip and 2100 Bioanalyzer (Agilent Technologies, Santa Clara, CA, USA). Only samples with an RNA integrity score (RIN)≥8.5 were used. RNA-Seq was completed using the PAT-Seq approach with libraries constructed from 1 µg total RNA. Sequencing was conducted as described previously (Harrison et al., 2015), except that 150 base chemistry was used on a HiSeq1500 system (Illumina, Scoresby, Victoria, Australia). Raw read filtering, quality checking and alignment against the Ensembl human genome reference (version hg38, release 82) was completed using the Tail-Tools bioinformatics suite (https://github.com/Victorian-Bioinformatics-Consortium/tail-tools). The data are available through the NCBI Gene Expression Omnibus (GEO) (https://www.ncbi.nlm.nih.gov/gds/) with accession number GSE101745.

### Bioinformatics analyses

Differentially expressed genes were called by the in-house Tail-Tools pipeline, except in the case of GSEA, where raw gene-wise counts from Tail-Tools were first analysed using the Limma-voom approach (Ritchie et al., 2015). Any visualizations of RNA-Seq data were generated from raw genewise counts that were variance stabilized by the ‘varistran’ R package (https://github.com/MonashBioinformaticsPlatform/varistran). GSEA was applied using Camera (Wu and Smyth, 2012), with gene ontology (GO) gene sets taken from the MSigDB Molecular Signatures Database (http://software.broadinstitute.org/gsea/msigdb/) (Subramanian et al., 2005). To obtain breast cancer data from TCGA, raw gene expression counts and patient information were downloaded using the GDC data portal (https://portal.gdc.cancer.gov/). TNBCs were selected based on clinical immunohistochemical data and divided by stage. TCGA gene expression analyses were conducted using the Limma-voom approach and GSEA was performed as for RNA-Seq data. CpG islands were discovered using the web-based Softberry CpGFinder (www.softberry.com).

### Single nucleotide polymorphism arrays

Genomic DNA (500 ng) was analysed using Infinium™ HumanCytoSNP-12 v2.1 300K BeadChip arrays (Illumina). After processing data in GenomeStudio (Illumina), SNP allelic ratios and copy number intensity were imported into Nexus Copy Number v8.0 (BioDiscovery, CA, USA). Data were normalised to diploid regions and segmented using the SNP-FASST2 segmentation algorithm, with a *P*-value of <0.5×10^−8^ and a minimum number of probes of 10. Copy number gain and loss were called at log_2_ ratios of +0.1 and −0.1, respectively. Allelic imbalance and loss of heterozygosity were called at B allele frequencies of 0.4 and 0.8, respectively. Concordance between cell line pairs was calculated as the percentage of base pairs with a different copy number state divided by the total number of base pairs included in the segmentation analysis.

### Histology

Tissues (one large lobe from liver and lung per mouse) were fixed overnight using 10% neutral buffered formalin and then sectioned (3 µm) for haematoxylin and eosin staining. Slides were scanned using an Aperio ScanScope with ImageScope software (Leica Microsystems, Macquarie Park, NSW, Australia) or an Olympus VS120 microscope and scanner with OlyVIA software (Olympus, Notting Hill, Victoria, Australia).

### 3D *in vitro* proliferation assays

For 3D assays, cells (2000/well) were seeded onto bovine type I collagen (2.5 mg/ml) gels (PureCol, Advanced BioMatrix, Carlsbad, CA, USA) prepared in white 96-well tissue culture plates. The luciferase-based Cell Titer Glo protocol (Promega) was used in accordance with the manufacturer's instructions and a 1.0 s luminometer integration time.

### *In vitro* analysis of cell migration

Transwell migration and invasion assays were conducted as described previously (Johnstone et al., 2015). Briefly, 2×10^5^ cells were seeded into the upper chamber of Fluoroblok (8 µm membrane pore size) inserts (Corning Life Sciences, Oneonta, NY, USA) in serum-free medium. Cells were allowed to migrate for 6.5 h toward 10% serum-containing medium (700 µl) in the base of the unit. Inserts were incubated with 4 µg/ml calcein AM (Enzo Biochem Inc., New York, NY, USA) in PBS prior to imaging and counting of cells.

### Monitoring of tumour growth and metastasis *in vivo*

NOD.Cg-*Prkdc^scid^ Il2rg^tm1Wjl^*/SzJ (NSG) immunodeficient mice (*Mus musculus*, obtained from Walter and Eliza Hall Institute of Medical Research, Parkville, Victoria, USA) were maintained in a specific pathogen-free environment and fed *ad libitum*. All procedures involving mice conformed to National Health and Medical Research Council animal ethics guidelines and were approved by the Animal Experimentation and Ethics Committee (AEEC) of the Peter MacCallum Cancer Centre. Mammary tumours were established orthotopically in the right-side inguinal mammary gland of 6- to 8-week-old female mice, and monitored for growth and surgically resected as described previously (Johnstone et al., 2015). Briefly, viable tumour cells resuspended in PBS were mixed with 10-30% Cultrex (Trevigen) and inoculated (1×10^6^ cells) into the right-side inguinal mammary gland of female mice and then surgically resected at a size of 200-400 mm^3^. Tumour size was measured using callipers and tumour volumes were estimated by the modified ellipsoidal formula: volume=1/2(length×width^2^) (Tomayko and Reynolds, 1989). Differences in primary tumour growth rates were calculated using mixed-effects linear regression modelling as previously described (Johnstone et al., 2015). Primary tumour growth and metastasis were also monitored by *in vivo* bioluminescence imaging as described previously (Johnstone et al., 2008) using intraperitoneal injection of *in vivo* grade D-luciferin substrate (VivoGlo, Promega) and a Xenogen Lumina II *in vivo* imaging system (Caliper Life Sciences, Perkin Elmer, Hopkinton, MA, USA).

## Supplementary Material

Supplementary information
