# Supplementary material for: Functional and genomic characterisation of a xenograft model system for the study of metastasis in triple-negative breast cancer
Source: Dis Model Mech. 2018 May 29;11(5):dmm032250. doi: 10.1242/dmm.032250 (PMC5992606; doi:10.1242/dmm.032250)
Supplement: Supplementary information [file dmm-11-032250-s1.pdf]

SUPPLEMENTARY FIGURES

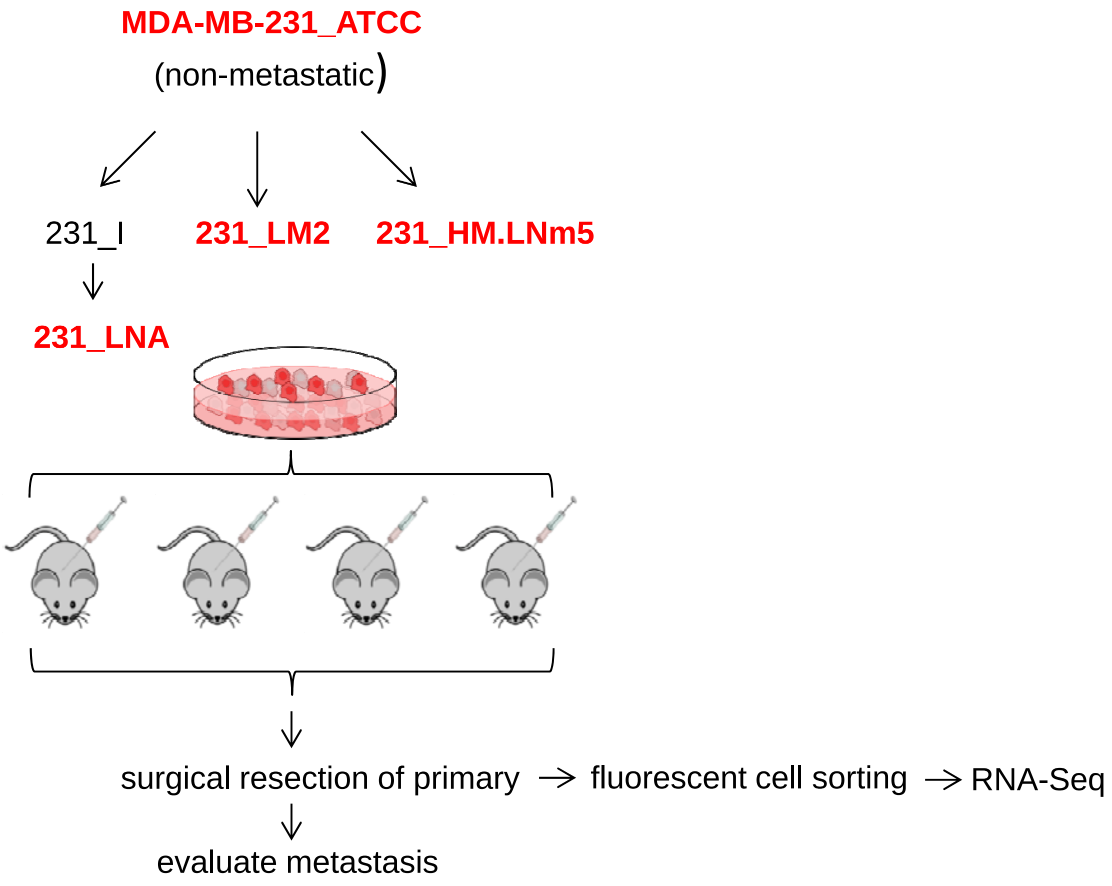

Figure S1: The study work flow. The 231_LNA, 231_LM2 and 231_HM.LNm5 cell lines were all originally derived from non-metastatic parental MDA-MB-231_ATCC (231_ATCC) cells. The 231_I cell population was derived from late-passage *in vitro* cultured MDA-MB-231_ATCC cells (see Materials and Methods). Each of the 4 cell lines were inoculated into the mammary glands of female NSG mice. The resulting primary tumours were surgically resected and sorted for eGFP and/or tdTomato expression by flow cytometry. Total RNA was isolated from sorted primary tumour cells for digital RNA sequencing (RNA-Seq) (Harrison et al., 2015). The same mice were subsequently monitored for the development tumour metastasis by *in vivo* bioluminescent imaging and this was quantified by analysis of secondary organs at harvest.

Disease Models & Mechanisms • Supplementary information

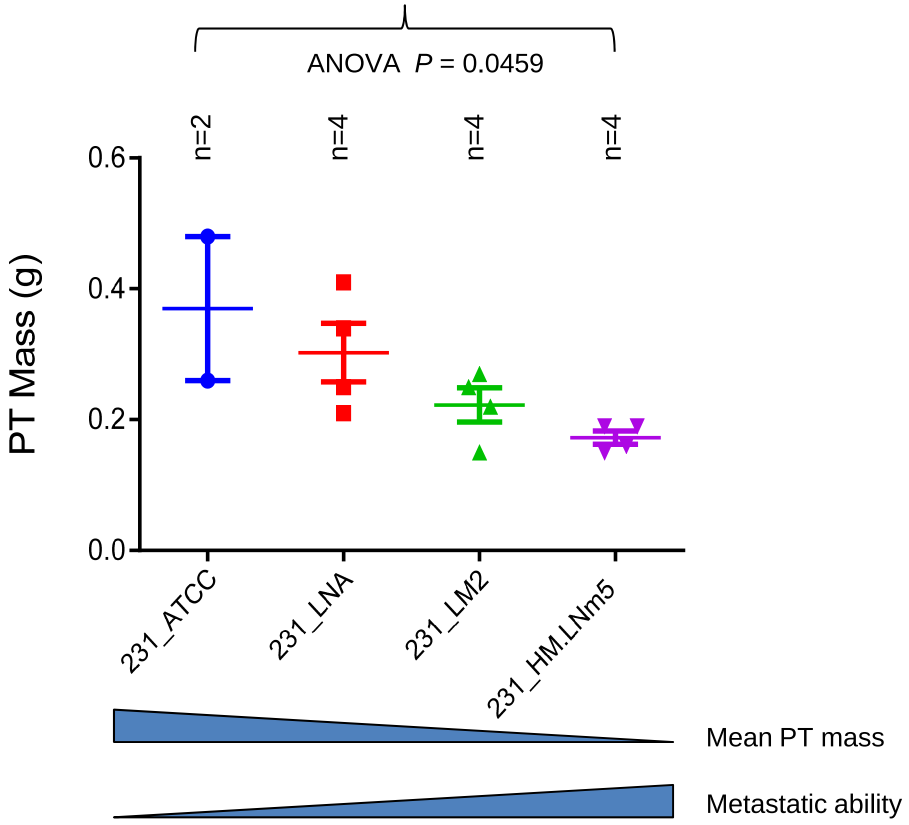

Figure S2: Mass of resected primary tumours. 231_LM2 (n=4) and 231_HM.LNm5 (n=4) primary tumours were surgically resected at day 18, 231_LNA (n=4) at day 21, and 231_ATCC (n=2) at day 72 after inoculation. Mean +/- S.E.M.

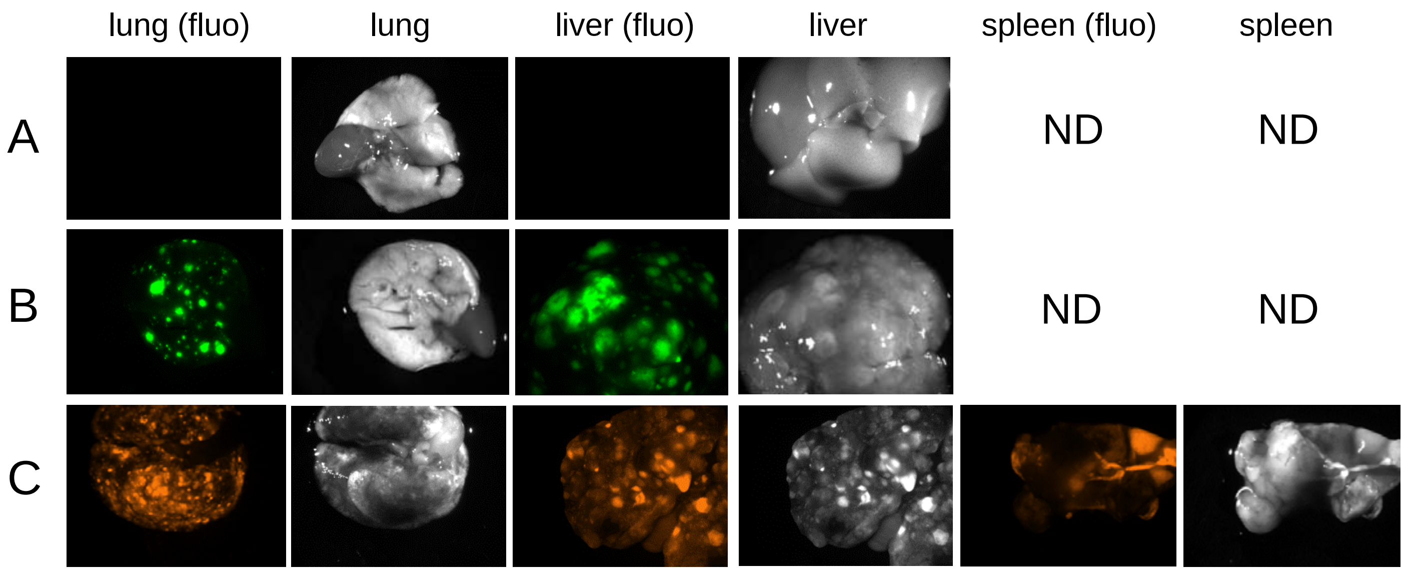

Figure S3: Spontaneous metastatic capacity of the 231_ATCC, 231_LNA, and 231_HM.LNm5 breast cancer models to lung, liver and spleen in NSG mice. Paired fluorescent (fluo) (left panels) and greyscale (right panels) images of representative whole mouse lungs, livers and spleen at harvest, 22 days after primary tumour resection. **A.** 231_ATCC (tdTomato fluorescence, pseudocoloured green). **B.** 231_LNA (tdTomato fluorescence, pseudocoloured green). **C.** 231_HM.LNm5 (tdTomato fluorescence, pseudocoloured orange). Metastatic lesions were not identified in the 231_ATCC model. N/D, not done. Images were captured using a fluorescent dissecting stereomicroscope (Olympus) and SPOT digital camera (Diagnostic Instruments, Inc.). Magnification 7x.

Disease Models & Mechanisms • Supplementary information

A

LN met	231ATCC	231LNA	231LM2	231HM
**Yes**	0	0	1	3
**No**	4	4	3	1

P = 0.0459

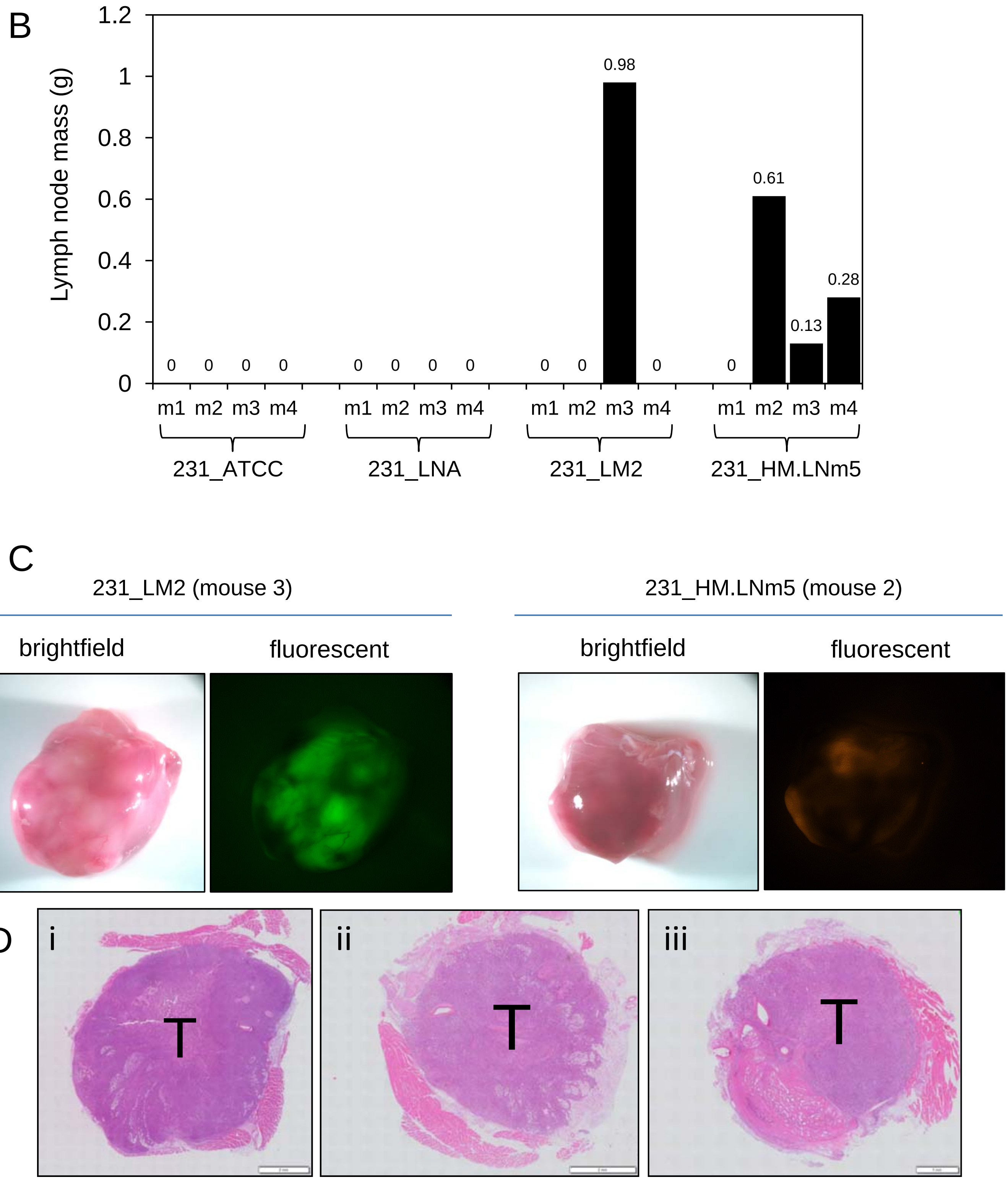

Disease Models & Mechanisms • Supplementary information

Figure S4: Spontaneous metastasis of MDA-MB-231 variants to lymph node in NSG mice. A. Incidence of ipsilateral axillary lymph node (LN) metastasis in the four isogenic xenograft models at harvest (n = 4 for each line). $P < 0.05$ (Fisher's Exact Test),. **B.** Mass (g) of ipsilateral axillary lymph node metastases in the four different models at harvest. **C.** Brightfield (left panels) and fluorescent (right panels) images of representative ipsilateral axillary lymph node metastases from the 231_LM2 (left side, GFP fluorescence) and 231_HM.LNm5 (right side, tdTomato fluorescence) tumour lines. Magnification 7x. **D.** Representative H&E stained sections (3μM) from the three ipsilateral lymph node metastases formed by the 231_HM.LNm5. Scale bars are shown at the bottom right **i-ii.** 2mm. **iii.** 1mm.

Supp. Figure 5A: 231_ATCC

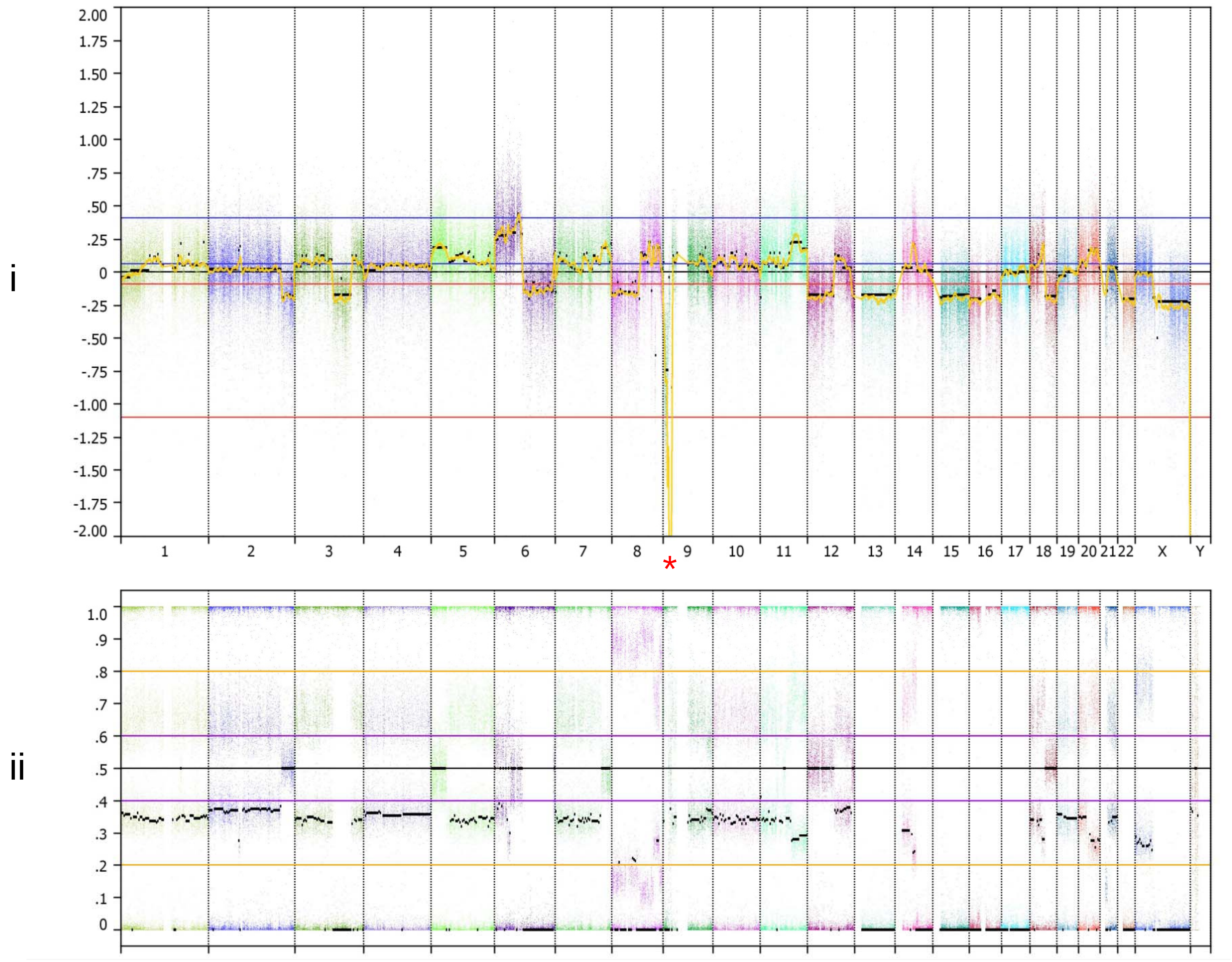

Disease Models & Mechanisms • Supplementary information

Supp. Figure 5B: 231_I

i

ii

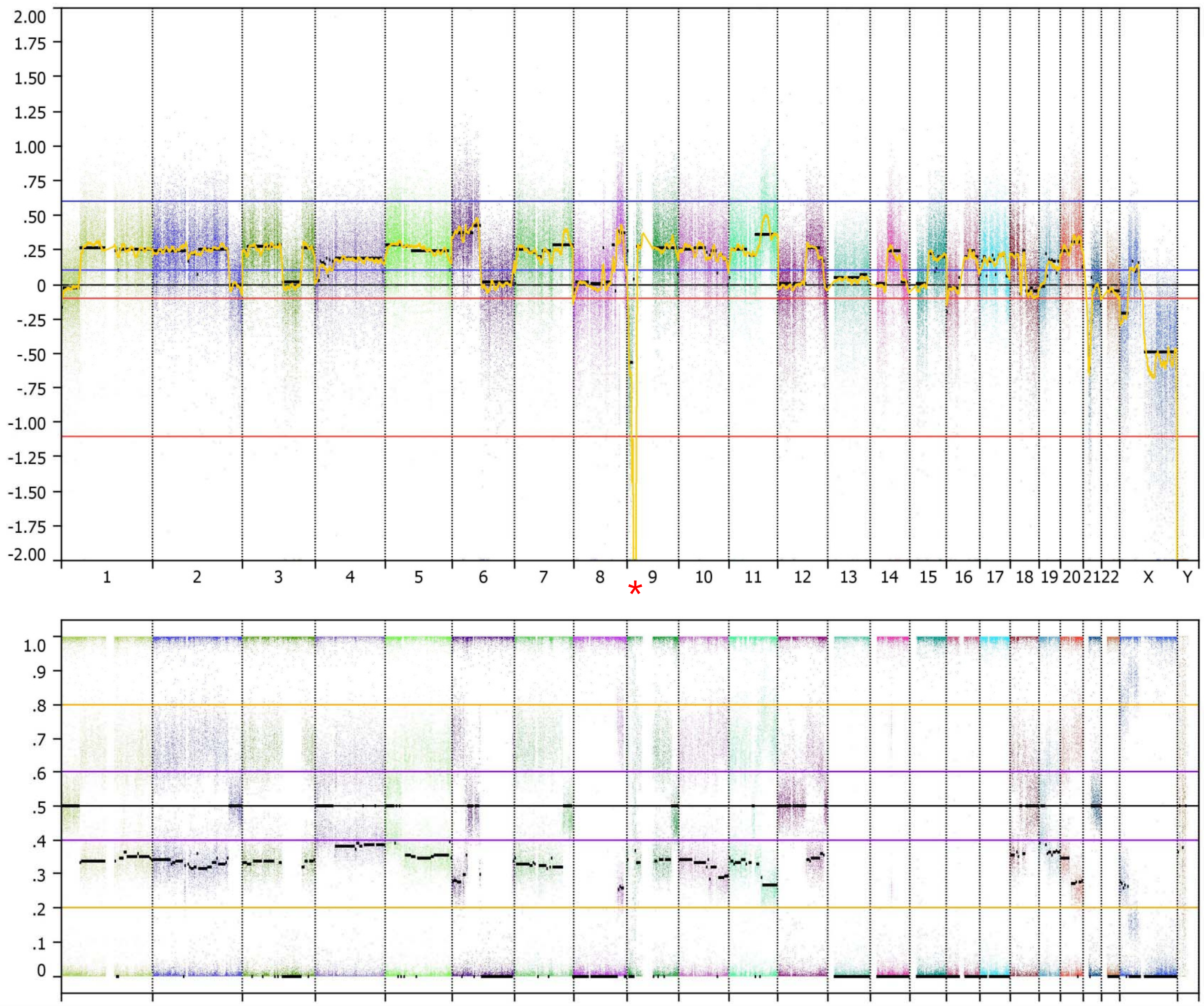

Disease Models & Mechanisms • Supplementary information

Supp. Figure 5C: 231_LM2

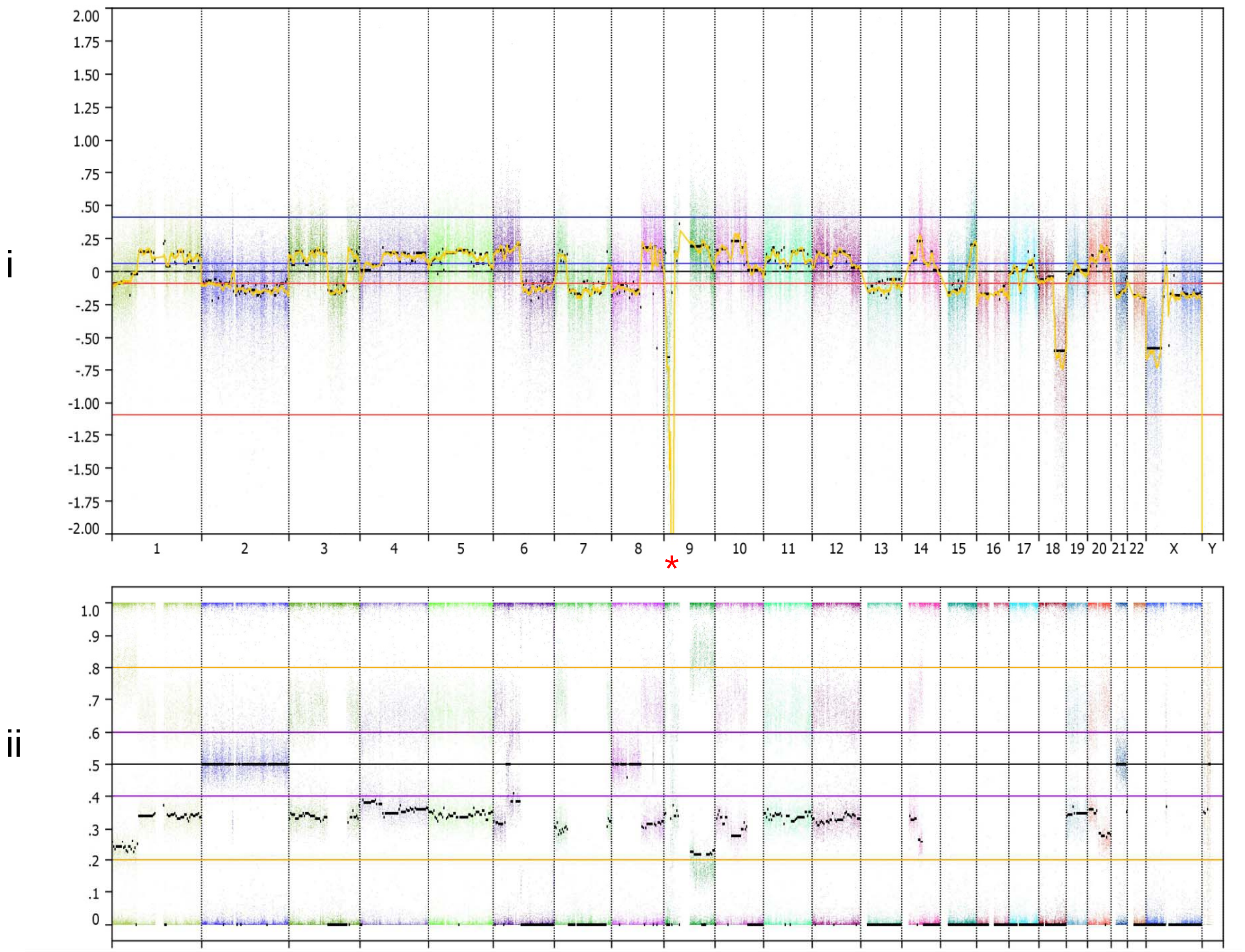

Disease Models & Mechanisms • Supplementary information

Supp. Figure 5D: 231_HM

i

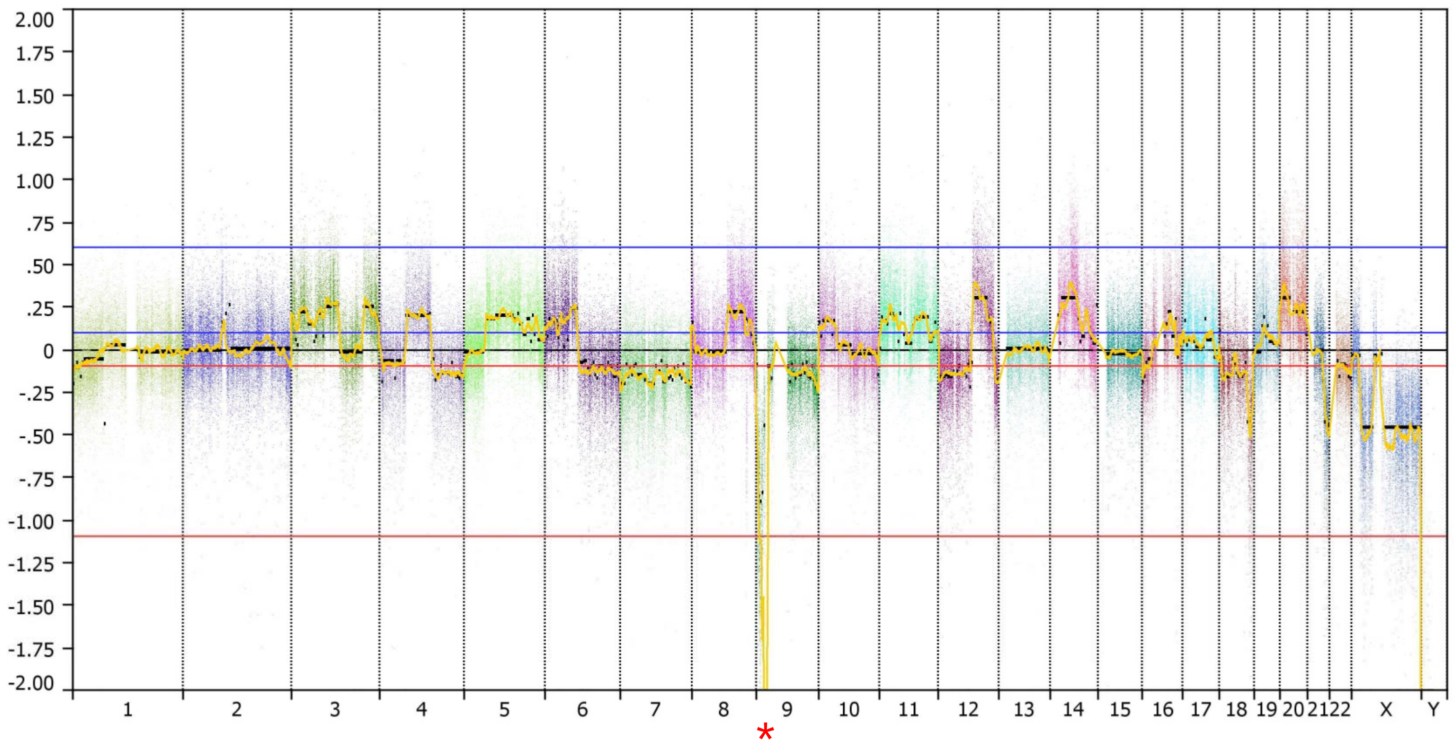

ii

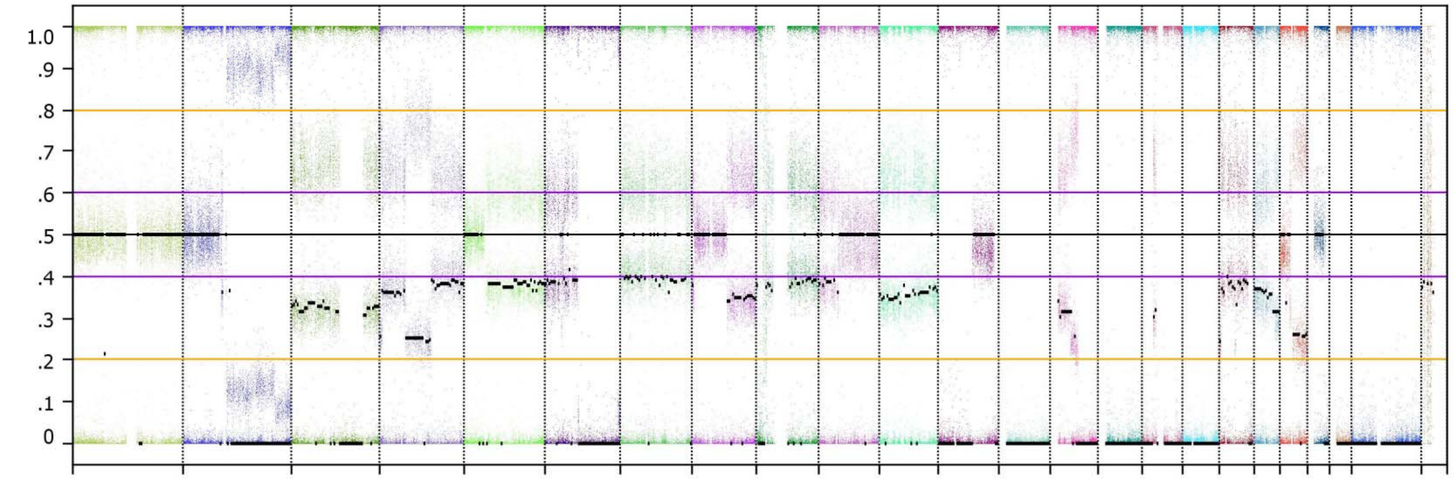

Disease Models & Mechanisms • Supplementary information

Figure S5: DNA copy number variation and allelic imbalance in four MDA-MB-231 variant cell lines. Raw Infinium™ HumanCytoSNP-12 v2.1 300K BeadChip data was processed for each cell line as described in Materials and Methods. **A.** 231_ATCC. **B.** 231_I. **C.** 231_LM2. **D.** 231_HM. **i.** Genome-wide CNVs (+2.00 max. to -2.00 min.) versus chromosomal location were plotted for each cell line in a linear manner. Horizontal red lines represent thresholds for copy number loss (log_2 ratio < -0.2) and homozygous deletion (log_2 ratio < -1). Horizontal blue lines represent thresholds for copy number gain (log_2 ratio > 0.2) and amplification (log_2 ratio > 0.6). The yellow line represents a moving average of copy number ratio. Black lines represent copy number segments. **ii.** B allele frequency (y-axis) versus chromosomal location (x-axis) was plotted. Horizontal yellow lines represent threshold for loss of heterozygosity (B allele frequency of < 0.2 or > 0.8). Horizontal purple lines represent threshold for allelic imbalance (B allele frequency > 0.6 or < 0.4). Black lines represent called B allele frequency segments.

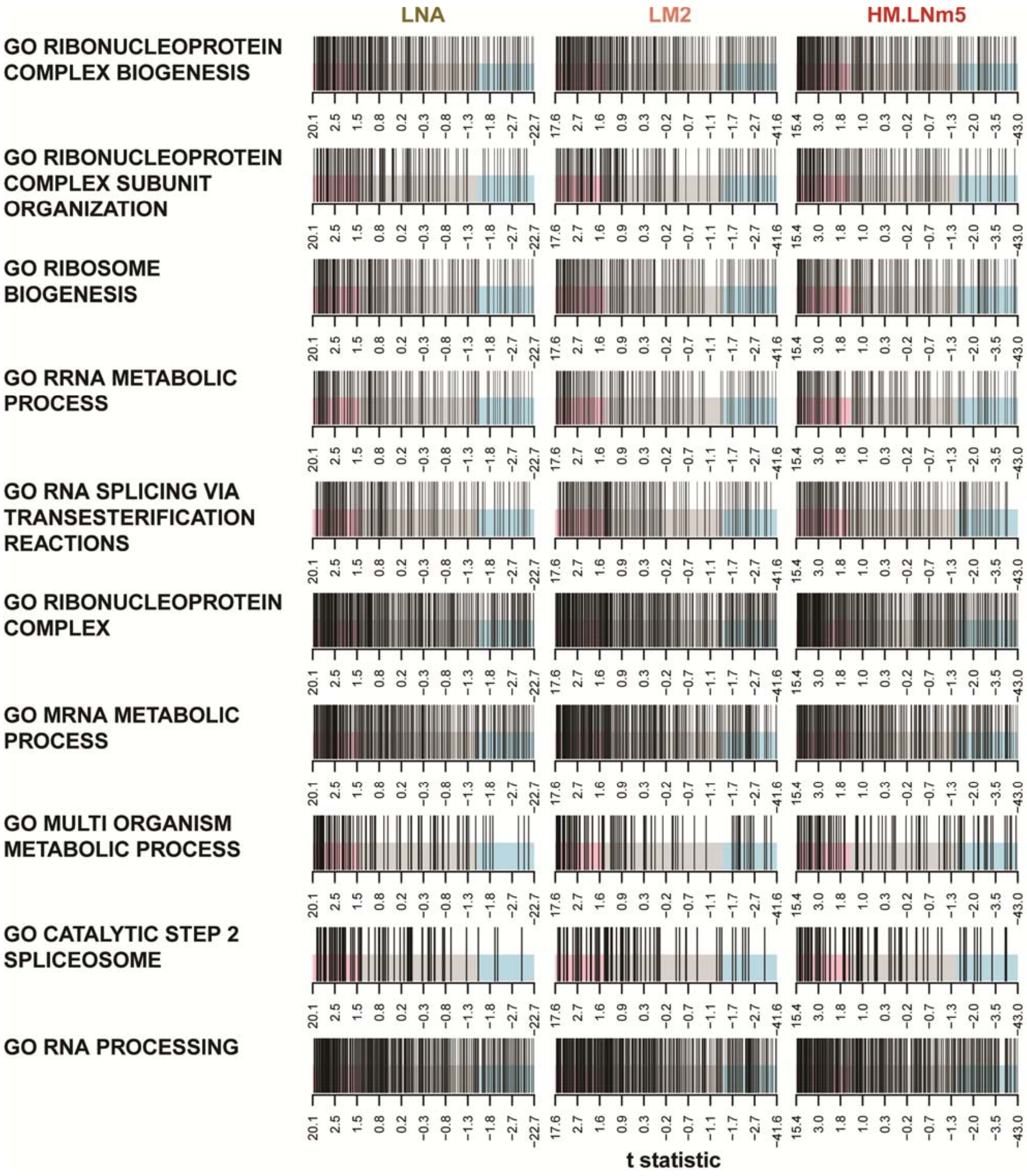

Figure S6: Bar plots evaluating individual genes from the gene sets significantly upregulated (FDR < 0.05) in each of the three metastatic models versus the non-metastatic 231_ATCC model (see Table 1). Each vertical bar represents one gene within the gene set. Upregulated genes have a positive t-statistic whereas down regulated genes have a negative t-statistic.

Disease Models & Mechanisms • Supplementary information

A

Gene	RNA-Seq data			Adjusted *P* (ANOVA)
	231_LNA	231_LM2	231_HM.LNm5	
Vimentin	1.45	0.98	0.93	0.318

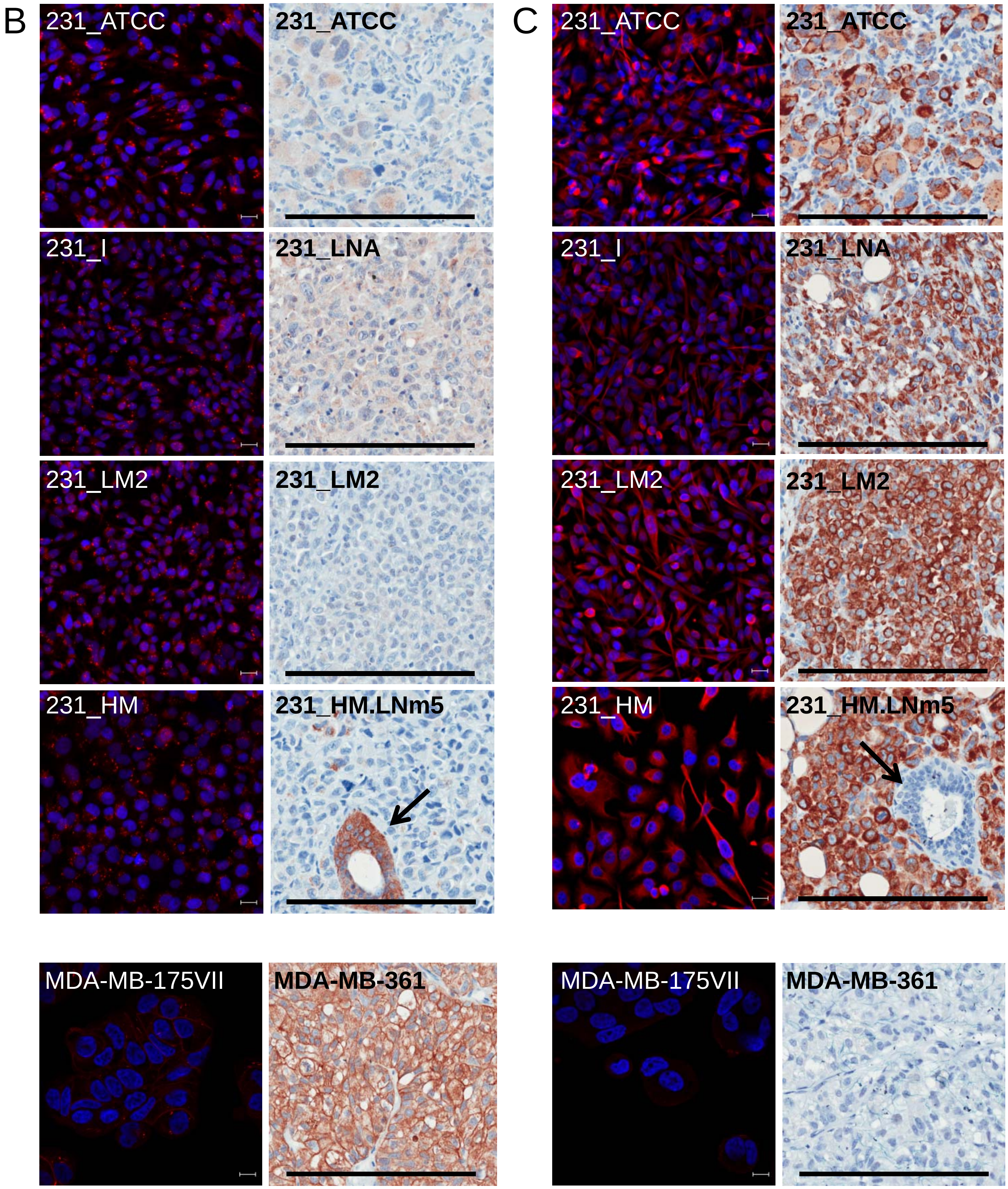

Disease Models & Mechanisms • Supplementary information

Figure S7: Key markers of EMT are not dysregulated across four MDA-MB-231 derived breast tumour models. A. Expression of vimentin mRNA in tumour cells isolated from primary MDA-MB-231 mammary xenografts. Expression levels relative to non-metastatic 231_ATCC tumour cells were derived from RNA-Seq data. Vimentin mRNA was not differentially expressed ($P > 0.05$). E-cadherin mRNA was not detected in tumour cells by RNA-Seq. **B.** Distribution of E-cadherin protein in MDA-MB-231 variants using confocal immunofluorescence microscopy (left panels) or immunohistochemistry (right panels). Parental MDA-MB-231 lines lacking expression of fluorescent reporter genes were used for immunofluorescence as indicated (with the exception of 231_LM2 which expresses GFP). E-cadherin was absent from cell-cell contacts, however large intracellular aggregates were detected in each of the four cell lines, which could represent cross-reactivity with intracellular pools of P-cadherin or another closely related cadherin, since E-cadherin protein is absent from MDA-MB-231 cells (Rhys et al., 2017). Luminal breast cancer cell line MDA-MB-175VII was used as a positive control for E-cadherin expression. Similarly, E-cadherin was absent from cell-cell junctions in primary MDA-MB-231 derived orthotopic xenografts (right panels). Normal mouse mammary ductal epithelium (arrow) and luminal MDA-MB-361 breast cancer xenograft were used as positive controls for E-cadherin expression. **C.** Distribution of vimentin protein in MDA-MB-231 variants using confocal immunofluorescence microscopy (left panels) or immunohistochemistry (right panels). Parental MDA-MB-231 lines lacking expression of fluorescent reporter genes were used as indicated (with the exception of 231_LM2). A filamentous cytoplasmic staining pattern was observed for vimentin in each of the four models. Luminal breast cancer cell line MDA-MB-175VII was used as a negative control for vimentin expression. Vimentin was also strongly expressed in the cytoplasm in each of the four primary MDA-MB-231 derived orthotopic xenografts (right panels). Normal mouse mammary ductal epithelium (arrow) and luminal MDA-MB-361 breast cancer xenograft were used as negative controls for vimentin expression. White scale bars in immunofluorescence images (bottom right) represent 10μM for MDA-MB-231 images and 20μM for MDA-MB-175VII images (**B, C** left panels). Black scale bars in images from immunohistochemical analyses (**B, C** right panels) represent 200μm.

Disease Models & Mechanisms • Supplementary information

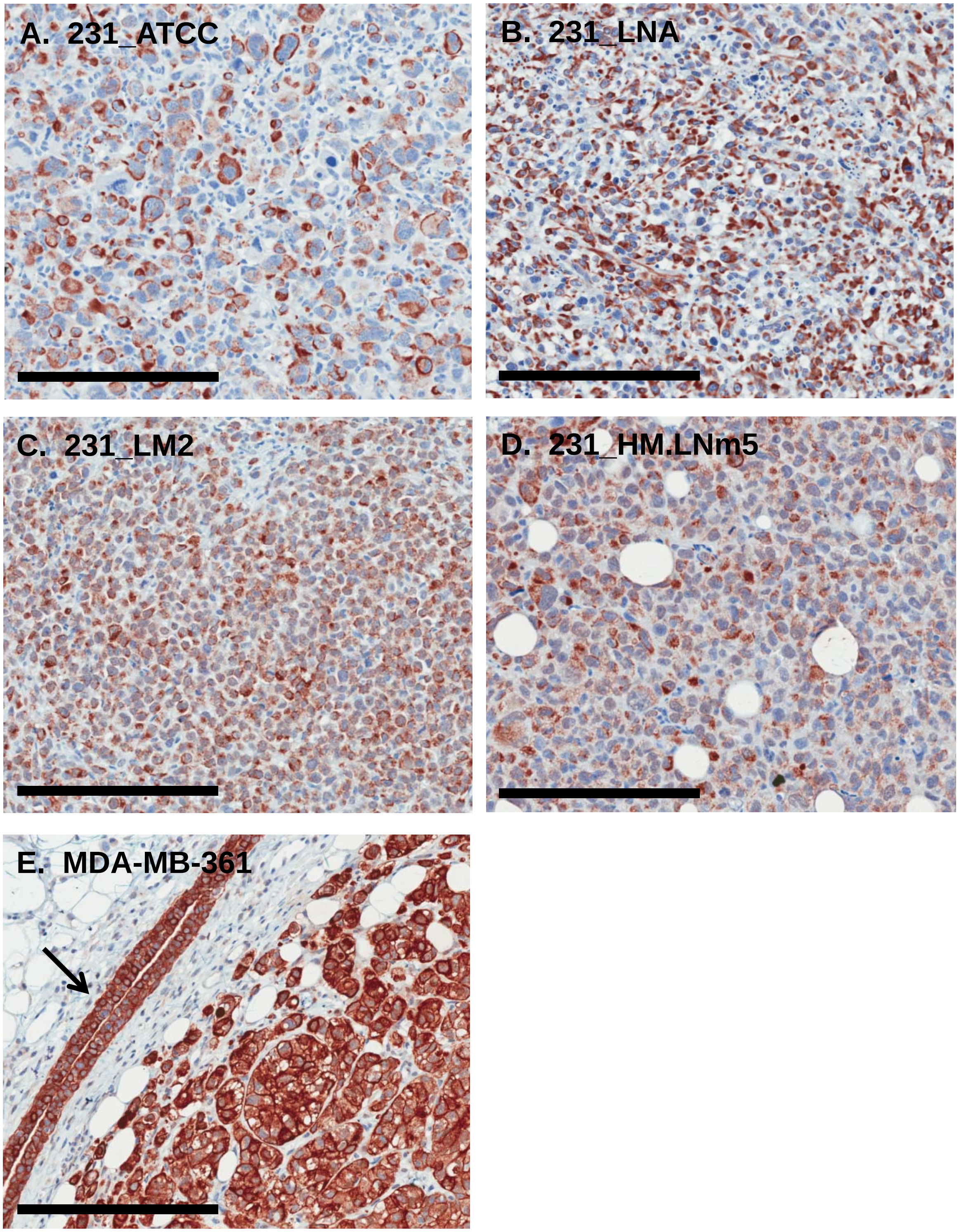

Figure S8: Immunohistochemical analysis of pan-cytokeratin expression in four MDA-MB-231 derived tumour models. Primary orthotopic xenografts of the four indicated MDA-MB-231 derived tumour models (**A-D**) were stained with a pan-cytokeratin antibody capable of recognising cytokeratins 1, 5, 6, and 8. A heterogeneous staining pattern of moderate intensity was observed in each tumour model. Mouse mammary ductal epithelium (arrow) and luminal (cytokeratin 8 positive) MDA-MB-361 xenograft (**E**) were used as positive controls. Black scale bars represent 200μm.

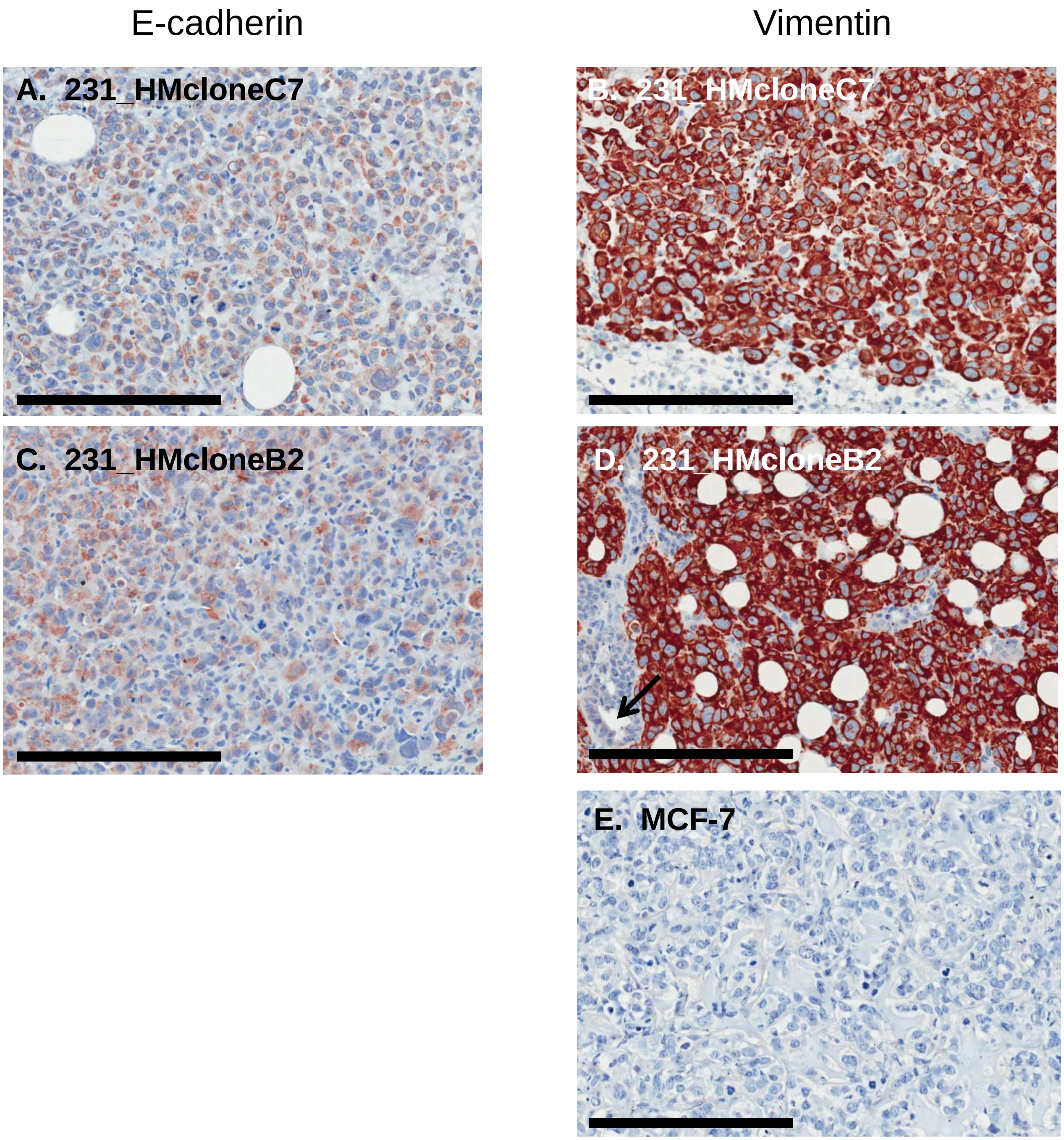

Figure S9: Immunohistochemical analysis of E-cadherin and vimentin protein expression and distribution in orthotopic xenografts formed by MDA-MB-231HM derived clonal daughter lines. 231_HMcloneC7 and 231_HMcloneB2 are different single cell clones of parental 231_HM cells with different ploidy (**see Figure 4**) but a similar metastatic capacity (data not shown). Both lines were negative for E-cadherin (**A, C**) but strongly positive for cytoplasmic vimentin (**B, D**). Mouse mammary ductal epithelium (arrow, **D**) and luminal MCF-7 orthotopic xenograft (**E**) were used as negative controls for vimentin staining. Black scale bars represent 200µm.

Disease Models & Mechanisms • Supplementary information

A. Up regulated genes versus 231_ATCC

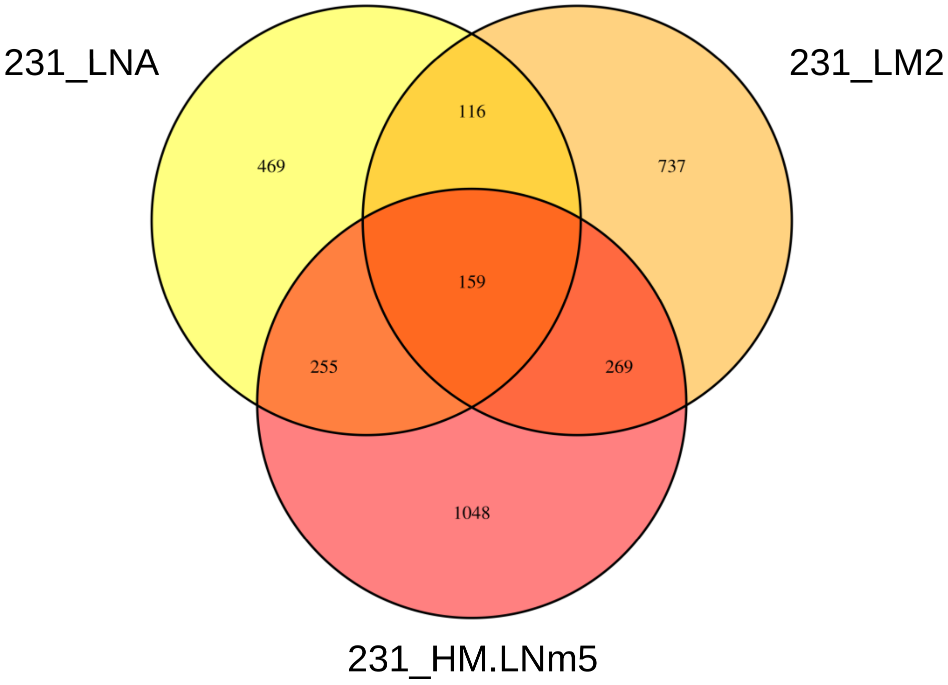

B. Down regulated genes versus 231_ATCC

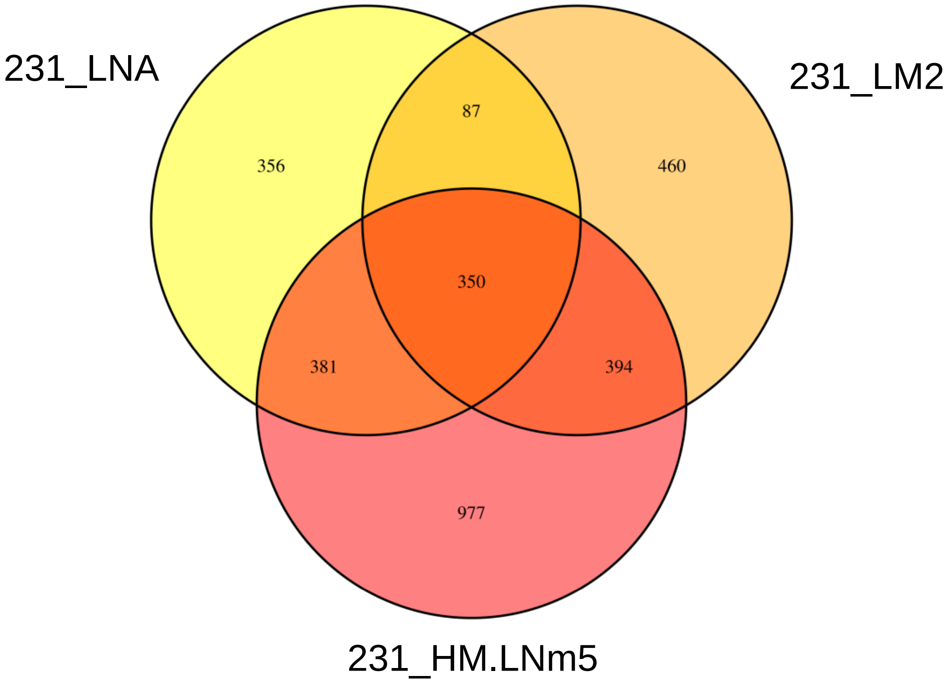

Disease Models & Mechanisms • Supplementary information

Figure S10: Differential gene expression across four MDA-MB-231 xenograft models visualised using Venn diagrams. The RNA-Seq data from **Table S3** are presented here as Venn diagrams. **A.** Genes up regulated in the three metastatic models versus the non-metastatic 231_ATCC line. **B.** Genes down regulated in the three metastatic models versus the non-metastatic 231_ATCC line.

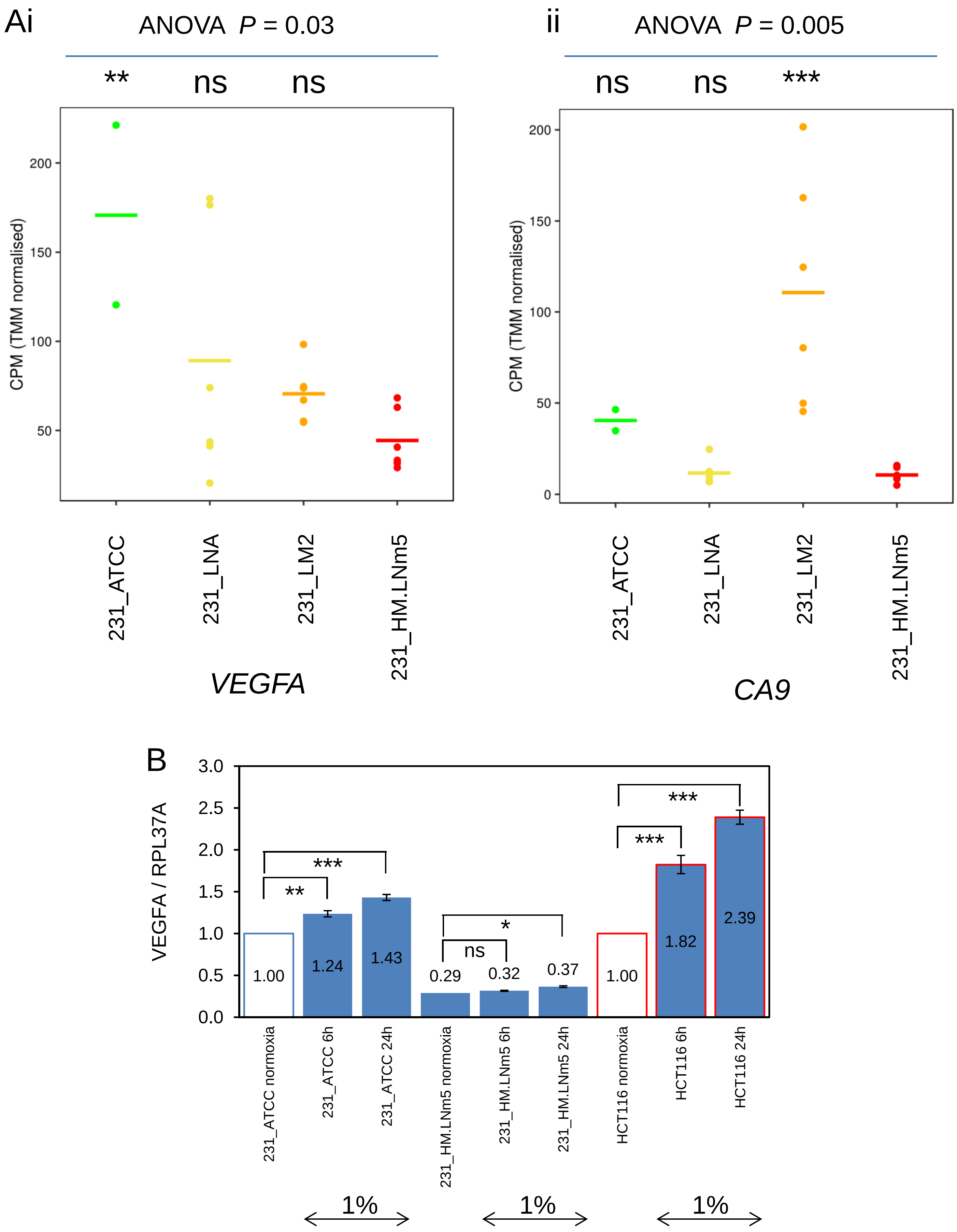

Disease Models & Mechanisms • Supplementary information

Figure S11: A. Expression levels (RNA-Seq TMM normalised tag count) of vascular endothelial growth factor-A (*VEGFA*, **i**) and carbonic anhydrase-9 (*CA9*, **ii**) in cancer cells isolated from 231_ATCC (n=2), 231_LNA (n=6), 231_LM2 (n=6) and 231_HM.LNm5 (n=6) mammary xenografts. Expression levels were compared by one-way ANOVA followed by Dunnett's post-hoc test for multiple comparisons. ** $P < 0.01$, 231_ATCC vs 231_HM.LNm5. *** $P < 0.001$, 231_LM2 vs 231_HM.LNm5. ns, not significant vs. 231_HM.LNm5. **ii.** VEGFA mRNA levels were analysed by TaqMan qRT-PCR in 231_ATCC and 231_HM.LNm5 cell lines cultured in 2D under normoxic conditions or in 1% oxygen for 6h or 24h as indicated. VEGFA expression in 231_ATCC cells cultured under normoxia was set to 1 as indicated. HCT116 colorectal cancer cells were included as a positive control for VEGFA induction by hypoxia. Mean +/- S.D. n=3. Data were analysed by Student's t-test. * $P < 0.05$, ** $P < 0.01$, *** $P < 0.001$ versus corresponding normoxic control. ns, not significant.

References for Supplementary Figure Legends

Cailleau, R., Mackay, B., Young, R. K. and Reeves, W. J., Jr. (1974). Tissue culture studies on pleural effusions from breast carcinoma patients. *Cancer Res.* **34**, 801-9.

Chang, X. Z., Li, D. Q., Hou, Y. F., Wu, J., Lu, J. S., Di, G. H., Jin, W., Ou, Z. L., Shen, Z. Z. and Shao, Z. M. (2007). Identification of the functional role of peroxiredoxin 6 in the progression of breast cancer. *Breast Cancer Res.* **9**, R76.

Fietz, E. R., Keenan, C. R., Lopez-Campos, G., Tu, Y., Johnstone, C. N., Harris, T. and Stewart, A. G. (2017). Glucocorticoid resistance of migration and gene expression in a daughter MDA-MB-231 breast tumour cell line selected for high metastatic potential. *Sci. Rep.* **7**, 43774.

Harrison, P. F., Powell, D. R., Clancy, J. L., Preiss, T., Boag, P. R., Traven, A., Seemann, T. and Beilharz, T. H. (2015). PAT-seq: a method to study the integration of 3'-UTR dynamics with gene expression in the eukaryotic transcriptome. *RNA* **21**, 1502-10.

Johnstone, C. N., Mongroo, P. S., Rich, A. S., Schupp, M., Bowser, M. J., Delemos, A. S., Tobias, J. W., Liu, Y., Hannigan, G. E. and Rustgi, A. K. (2008). Parvin-beta inhibits breast cancer tumorigenicity and promotes CDK9-mediated peroxisome proliferator-activated receptor gamma 1 phosphorylation. *Mol. Cell. Biol.* **28**, 687-704.

Johnstone, C. N., Smith, Y. E., Cao, Y., Burrows, A. D., Cross, R. S., Ling, X., Redvers, R. P., Doherty, J. P., Eckhardt, B. L., Natoli, A. L. et al. (2015). Functional and molecular characterisation of EO771.LMB tumours, a new C57BL/6-mouse-derived model of spontaneously metastatic mammary cancer. *Dis. Model. Mech.* **8**, 237-51.

SUPPLEMENTARY TABLES

Table S1: The retroviral vectors used for tagging and sorting MDA-MB-231 primary tumours. The 231_ATCC line represents early passage MDA-MB-231 obtained from ATCC (Cailleau et al., 1974). The late-passage 231_I line was originally obtained from Dr. Gregory Hannigan, Hospital for Sick Kids, Toronto, Canada (Johnstone et al., 2008). The pFB_neo_GFP retroviral vector was a kind gift from Hiroshi Nakagawa. The pBabe_puro_Fluc vector was a kind gift from Wafik El-Deiry. The pBabe_Bla_tdTomato vector was described previously (Johnstone et al., 2008). 231_ATCC and 231_I cells were tagged with the indicated GFP, tdTomato, and Firefly luciferase expressing vectors (Johnstone et al., 2008). Reporter gene-tagged 231_I cells were inoculated orthotopically into the right-side inguinal mammary gland of a BALB/c-SCID mouse and a primary tumour grown. The 231_LNA line was isolated from a spontaneous ipsilateral axillary lymph node metastasis and retains expression of GFP, tdTomato, and Firefly luciferase. The GFP- and Firefly luciferase-expressing lung-homing 231_LM2 variant (also designated clone 4175) was a kind gift from Joan Massague (Minn et al., 2005; Ponomarev et al., 2007). 231_LM2 was transduced with pBabe_Bla_tdtomato, selected with Blasticidin S, and sorted for tdTomato expression using flow cytometry (FACSAria, Beckton Dickinson), yielding 231_LM2tom. The 231_HM variant was a kind gift from Z-L. Ou and Z-M. Shao, Fudan University, Shanghai, China (Chang et al., 2007). 231_HM were transduced with Firefly luciferase-expressing retrovirus and then inoculated into the right-side inguinal mammary gland of a BALB/c-SCID mouse and primary tumours allowed to form. A spontaneous ipsilateral axillary lymph node metastasis was isolated, expanded *ex vivo*, and subsequently transduced with tdTomato expressing retrovirus. The cells were sorted for tdTomato expression by flow cytometry, which yielded the 231_HM.LNm5 line, which has also been described previously (Fietz et al., 2017).

Model	Precursor line	GFP vector	tdTomato vector	Luciferase Vector	PT sorted using	Ref
231_ATCC	23 _ATCC	pFB_neo_GFP	pBabe_Bla_tdtomato	pBabe_puro_Fluc	tdTomato	1
231_LNA	231_I	pFB_neo_GFP	pBabe_Bla_tdtomato	Babe_puro_Fluc	GFP & tdTomato	2
231_LM2tom	231_LM2	Δ45-TGL	p _Bla_tdtomato	Δ45-TGL	GFP	3,4
231_HM.LNm5	231_HM	nd	pBabe_Bla_tdtomato	pBabe_puro_Fluc	tdTomato	5,6

Disease Models & Mechanisms • Supplementary information

Table S2: Two-way comparisons of DNA copy number variations (CNVs) among MDA-MB-231 derived cell lines. Raw Infinium[TM] HumanCytoSNP-12 v2.1 300K BeadChip data was processed as described in Materials and Methods. DNA copy number gains and losses that were concordant or discordant in each of the two way comparisons were tallied and used to estimate the genetic difference between the three metastatic lines (231_I, 231_LM2, 231_HM) and non-metastatic parental 231_ATCC cells.

Comparison	Concordant gains	Concordant losses	Total concordant CNA	% genome CNA shared	Discordant gains	Discordant losses	Total discordant CNA	Genome bp assayed	% difference
**231_ATCCv231_I**	1738095319	33925410	**1772020729**	**62.8**	309879969	119993592	**429873561**	**2822801661**	**15.2**
**231_ATCCv231_LM2**	1435013235	44172722	**1479185957**	**52.4**	637053810	91475423	**728529233**	**2822303392**	**25.8**
**231_ATCCv231_HM**	620830286	21580091	**642410377**	**22.7**	1291504697	574146589	**1865651286**	**2824262004**	**66.1**

Disease Models & Mechanisms • Supplementary information

Table S3: Summary of RNA-Seq derived gene expression data (with FDR <0.01).

Sheet 1: Genes upregulated in metastatic cells (**metastasis initiation genes**) (i.e. upregulated in 231_LNA, 231_LM2 and 231_HM.LNm5 vs. 231_ATCC).

Sheet 2: Genes upregulated in highly metastatic cells only (**metastatic virulence genes**) (i.e. upregulated in 231_HM.LNm5 only)

Sheet 3: Genes down regulated in metastatic cells (**metastasis suppression genes**) (i.e. down regulated in 231_LNA, 231_LM2 and 231_HM.LNm5 vs. 231_ATCC)

Sheet4: Genes down regulated in highly metastatic cells only (**metastatic avirulence**) (i.e. down regulated in 231HM.LNm5 only).

FC, fold-change.

Click here to Download Table S3

Table S4: Genes deregulated in metastatic cells allocated to gene expression category (Nguyen and Massague, 2007). Candidate metastasis initiation genes (initiation) are genes significantly upregulated in all three metastatic models (231_LNA, 231_LM2, 231_HM.LNm5) versus the non-metastatic 231_ATCC model. **Candidate metastasis virulence genes** (virulence) are genes significantly upregulated specifically in the highly-metastatic 231_HM.LNm5 model versus the moderately metastatic (231_LNA, 231_LM2) models and non-metastatic 231_ATCC model. **Candidate metastasis suppressor genes** (suppression) are genes significantly down regulated in all three metastatic models (231_LNA, 231_LM2, 231_HM.LNm5) versus the non-metastatic 231_ATCC model. Finally, **candidate metastasis avirulence genes** (avirulence) are genes significantly down regulated specifically in the highly-metastatic 231_HM.LNm5 model versus the moderately metastatic (231_LNA, 231_LM2) models and non-metastatic 231_ATCC model. Expression of genes shaded grey were validated by qRT-PCR.

Class	Gene Symbol	Name	Class
**Metallothioneins**			
	MT1A	metallothionein 1A	Virulence
	MT1E	metallothionein 1E	Virulence
	MT1M	metallothionein 1M	Virulence
	MT2A	metallothionein 2A	Virulence
**Proteases / Cystatins**			
	CTSC	cathepsin C	Initiation
	ADAMTS1	ADAM metallopeptidase with thrombospondin type 1 motif 1	Virulence
	CST1	cystatin SN (type 2)	Suppression
	CST2	cystatin SA (type 2)	Suppression
	CST3	cystatin C (type 2)	Avirulence
	CST6	cystatin E/M (type 2)	Suppression
	CST4	cystatin S (type 2)	Suppression
	CSTP1	cystatin pseudogene 1	Suppression
**Integrins & Collagens**			
	ITGB4	integrin beta 4	Suppression
	COL18A1	collagen type XVIII alpha 1 chain	Avirulence
	COL6A1	collagen type VI alpha 1 chain	Avirulence
	COL6A2	collagen type VI alpha 2 chain	Avirulence
	COL13A1	collagen type XIII alpha 1 chain	Avirulence
**Potassium & Sodium channels**			
	KCNU1	potassium calcium-activated channel subfamily U member 1	Virulence
	KCNQ3	potassium voltage-gated channel subfamily Q member 3	Virulence
	KCNAB2	potassium voltage-gated channel subfamily A regulatory beta subunit 2	Initiation
	SCNN1A	sodium channel epithelial 1 alpha subunit	Suppression
**Immune Signaling**			
	CCL2	C-C motif chemokine ligand 2	Virulence
	IL1RL1	interleukin 1 receptor like 1	Virulence
	CXCR4	C-X-C motif chemokine receptor 4	Virulence
	PTPN7	protein tyrosine phosphatase, non-receptor type 7	Virulence
	CSF2RA	colony stimulating factor 2 receptor alpha subunit	Initiation
	CD33	CD33 molecule	Virulence
	CD74	CD74 molecule, major histocompatibility complex, class II invariant chain	Avirulence
**Vascular mimicry**			
	ENG	endoglin	Initiation
	TIE1	tyrosine kinase with immunoglobulin like and EGF like domains 1	Virulence
	SERPINE2	serpin family E member 2	Initiation
**BMP signaling**			
	BMP2	bone morphogenetic protein 2	Initiation
	BMP4	bone morphogenetic protein 4	Suppression
**LINC RNAs**			
	LINC00707	long intergenic non-protein coding RNA 707	Virulence
	LINC00880	long intergenic non-protein coding RNA 880	Virulence

Minn, A. J., Gupta, G. P., Siegel, P. M., Bos, P. D., Shu, W., Giri, D. D., Viale, A., Olshen, A. B., Gerald, W. L. and Massague, J. (2005). Genes that mediate breast cancer metastasis to lung. *Nature* **436**, 518-24.

Nguyen, D. X. and Massague, J. (2007). Genetic determinants of cancer metastasis. *Nat Rev Genet* **8**, 341-52.

Ponomarev, V., Doubrovin, M., Shavrin, A., Serganova, I., Beresten, T., Ageyeva, L., Cai, C., Balatoni, J., Alauddin, M. and Gelovani, J. (2007). A human-derived reporter gene for noninvasive imaging in humans: mitochondrial thymidine kinase type 2. *J. Nucl. Med.* **48**, 819-26.

SUPPLEMENTARY MATERIALS AND METHODS

Protein expression and distribution by immunofluorescence

Cells were cultured on 8-well plastic chamber slides (Nunc® Lab-Tek® Chamber Slide™ system, Sigma Aldrich), fixed with 4% paraformaldehyde for 30 min and permeabilized using 0.1% Triton X-100 for 5 min. Slides were blocked with 10% horse serum/1% bovine serum albumin (1h at room temperature). Mouse anti-human E-cadherin monoclonal antibody (clone 36, 1:100 dilution, BD Transduction Labs) or mouse anti-human vimentin monoclonal antibody (clone V9, 1:500 dilution, eBioscience, Thermo Fisher Scientific) were used with an Alexa-568 conjugated anti-mouse secondary antibody (Molecular Probes). Nuclei were visualised using DAPI (Sigma Aldrich) and images generated using a LSM780 inverted confocal microscope with a x10 objective and associated ZEN software (Zeiss, North Ryde, NSW, Australia).

Protein expression and distribution by immunohistochemistry

Orthotopic xenografts were resected from mouse mammary glands and fixed in 10% neutral buffered formalin for 4-8h. Heat-induced antigen retrieval was conducted for 15 min on 4μM sections submerged in citrate buffer (pH 6.0). Sections were blocked with 10% horse serum/1% bovine serum albumin (1h at room temperature) and then incubated overnight (4°C) in primary antibody. Mouse anti-human E-cadherin monoclonal antibody (clone 36, 1:100 dilution, BD Transduction Labs), mouse anti-human vimentin monoclonal antibody (clone V9, 1:500 dilution, eBioscience, Thermo Fisher Scientific), or mouse anti-human pan-cytokeratin monoclonal antibody (clone PCK-26, 1:300 dilution, Sigma Aldrich) were used. PCK-26 recognises an epitope on the type II cytokeratins 1, 5, 6, and 8. Sections were incubated with an HRP-conjugated anti-mouse secondary antibody (Dako, Agilent Technologies, Mulgrave, Vic, Australia) for 1h at room temperature prior to timed incubation with the chromogen 3,3'-Diaminobenzidine (Dako). Sections were counterstained with haematoxylin to visualise nuclei (blue) and slides were scanned using an Aperio Digital Pathology Slide Scanner (Leica Microsystems, Mt Waverley, Vic, Australia). Images were generated using Aperio ImageScope software (Leica Microsystems).
